# Breakfast Habits, Anthropometry, and Nutrition‐Related Outcomes in Adolescents From Low‐ and Middle‐Income Countries: A Systematic Review and Meta‐Analysis

**DOI:** 10.1002/cl2.70039

**Published:** 2025-04-22

**Authors:** Jordie A. J. Fischer, Jonathan Thomas, Despo Ierodiakonou, Kesso G. van Zutphen‐Küffer, Vanessa Garcia‐Larsen

**Affiliations:** ^1^ Sight and Life Basel Switzerland; ^2^ Texas A&M School of Medicine Bryan Texas USA; ^3^ Department of Primary Care and Population Health University of Nicosia Medical School Nicosia Cyprus; ^4^ Department of Human Nutrition & Health Wageningen University & Research Wageningen the Netherlands; ^5^ Program in Human Nutrition, Department of International Health Johns Hopkins University Baltimore Maryland USA

## Abstract

Breakfast skipping is a commonly reported dietary habit among adolescents despite this life stage marked by critical growth and development. Limited comparable evidence exists from low‐ and middle‐income countries (LMICs), where the detrimental effect of inadequate diets remains a major public health challenge. We conducted a systematic review to assess the scientific evidence available from LMICs regarding the association of breakfast skipping and consumption habits and anthropometry‐ and nutrition‐related outcomes in adolescents 10–19 years old. Electronic searches for relevant studies were conducted on MEDLINE, EMBASE, CINAHL, CENTRAL and Web of Science from the date of database inception until June 28, 2023. Additionally, reference lists of included studies and gray literature were searched. We included studies of all designs that compared breakfast skipping and consumption habits among adolescents aged 10–19 years in LMICs. Exclusion criteria included the following: review articles, if the target population age was outside the WHO definition of adolescents, assessed only lunch or dinner consumption, skipped any other meal besides breakfast, only collected point prevalence data, did not include a breakfast consumer control group, or co‐interventions were inconsistent across breakfast habit groups. The primary outcomes were body mass index (BMI in kg/m^2^), also defined categorically as underweight, normal weight, overweight and obese or as BMI‐for‐age (*z*‐score) and anemia (defined according to Hemoglobin (Hb) levels in different age groups for boys and girls). Secondary outcomes included other adiposity measures and nutrient concentrations or deficiencies. Title screening, data extraction, and risk of bias assessment were conducted in duplicate. The risk of bias was evaluated using the NHLBI Quality Assessment Tool for Observational Cohort and Cross‐Sectional Studies. Random‐effects meta‐analysis models were used to pool data for each outcome measure from the included studies. Standardized mean differences with 95% confidence intervals (95% CI) were calculated for continuous outcomes, while odds ratios (OR) with 95% CIs were computed for dichotomous outcomes. The certainty of the evidence for each outcome was appraised using the Grading of Recommendations Assessment, Development, and Evaluation (GRADE) approach. Our search yielded 3604 records, and 41 studies met our inclusion criteria. Among these, 39 cross‐sectional studies and two prospective cohort studies were eligible, with 36 providing data for meta‐analysis. Overall, there was very low certainty of evidence regarding the association between breakfast consumption habits and the risk of being overweight/obese, mainly due to the risk of bias and inconsistency. Adolescents who infrequently ate breakfast (0–2 days/week) were twice as likely to be overweight/obese (OR: 2.05, 95% CI: 1.61–2.61; *I* ^2^ = 85%; *n* = 15 studies) compared to regular breakfast consumers (5–7 days/week), while irregular breakfast consumers (3–4 days/week) had 32% higher likelihood of being overweight/obese (OR: 1.32, 95% CI: 1.16–1.50; *I* ^2^ = 59%; *n* = 9 studies). The odds of non‐daily breakfast consumers being overweight/obese were 38% higher than daily breakfast consumers (OR: 1.38, 95% CI: 1.19–1.59; *I* ^2^ = 54%; *n* = 10 studies). The odds of developing anemia were significantly higher for adolescents irregularly consuming breakfast compared to regular breakfast consumers (OR: 2.85, 95% CI: 1.71, 4.76; *I* ^2^ = 0%), with very low certainty of the evidence from two studies, limited by a very small sample size. Few studies reported on the association of breakfast skipping with other secondary adiposity outcomes (e.g., waist circumferences, waist‐to‐height ratio). We found very low certainty of evidence that breakfast skipping increases the risk of overweight/obesity and anemia, primarily derived from cross‐sectional studies. There is a paucity of evidence regarding breakfast habits and nutritional outcomes among adolescents in LMICs. Further cohort or intervention studies are warranted to elucidate the relationship between breakfast skipping and the risk of overweight/obesity, as well as other anthropometric and adiposity measurements within this demographic. Emphasis should also be placed on evaluating nutritional outcomes as a part of these assessments to better inform public health policy and programming best practices for adolescents to ensure the health and well‐being of the next generation. Breakfast integrated within school feeding programs may be well positioned as a double‐duty solution to tackle malnutrition in all its forms among adolescents.

## 1 Plain Language Summary

Breakfast skipping may increase the risk of overweight/obesity and anemia in adolescents in low‐ and middle‐income countries (LMICs), but more high‐quality studies assessing nutrition outcomes are needed.

Adolescence is a critical period of rapid growth and development, during which proper nutrition is vital. Malnutrition is a widespread issue among teenagers worldwide, especially in LMICs. Skipping breakfast is a common dietary habit among teens in LMICs. Our review suggests that breakfast skipping can lead to negative health outcomes, such as increasing adolescents' risk of being overweight or obese, as well as experiencing anemia or nutrient deficiencies.

### 1.1 The Review in Brief

Adolescents who infrequently consume breakfast are twice as likely to be overweight or obese compared to regular eaters. Adolescents who irregularly consume breakfast are more than twice as likely to have anemia compared to regular breakfast consumers.

### 1.2 What Is This Review About?

Breakfast skipping is a common dietary habit among teenagers, yet this life stage of important growth and development requires optimal nutrition. Scientific articles have reported many negative health consequences, such as overweight/obesity and anemia, of breakfast skipping in adolescents. However, these findings have never been synthesized specifically for adolescents in LMICs, who face unique health and nutrition challenges. As a result, the impact of breakfast skipping on the health and nutrition status of this population remains unclear.

### 1.3 What Is the Aim of This Review?

This Campbell systematic review aims to analyze the scientific evidence available from LMICs, regarding the relationship between breakfast habits, anthropometry (e.g., body mass index [BMI]), and nutrition‐related outcomes (e.g., anemia) in adolescents 10–19 years old. The review summarizes evidence from 41 observational studies, including 39 cross‐sectional and 2 prospective cohort studies.

### 1.4 What Are the Main Findings of This Review?

#### 1.4.1 What Studies Are Included?

All study designs evaluating the habit of skipping breakfast among adolescents aged 10–19 in LMICs were eligible for inclusion. This review included 41 studies, with 36 contributing to the quantitative meta‐analysis. These studies utilized various definitions of breakfast skipping, including frequency per week and yes/no categorization.

#### 1.4.2 Are Adolescent Breakfast Habits Associated With Obesity and Anemia?

In low‐ and middle‐income countries, adolescent breakfast skipping is associated with an increased likelihood of overweight and obesity. The likelihood of developing anemia differed between the breakfast habit groups, although only a limited number of studies reported on this association, as well as for other secondary outcomes, limiting the overall evidence.

### 1.5 What Do the Findings of This Review Mean?

Breakfast skipping may negatively contribute to the complex interplay of factors resulting in overweight and obesity. It may also be a risk factor for anemia, although our review indicates a very low certainty of evidence, and results should be interpreted cautiously. This review underscores the necessity for more research to be conducted on the health and nutrition implications of this widespread practice in the daily diets of adolescents globally.

### 1.6 How Up‐to‐Date Is This Review?

The review authors searched for studies up to June 2023. This Campbell Systematic Review was published in 2025.

## 2 Background

### 2.1 The Problem, Condition, or Issue

Our world is home to 1.8 billion adolescents aged 10–19 years worldwide, with 90% residing in LMICs (Progress for Children No. 10 and UNICEF 2012; United Nations 2017). Adolescent populations face a health and nutrition crisis due to the triple burden of malnutrition, characterized by the coexistence of undernutrition (wasting and/or stunting), micronutrient deficiencies, and overnutrition (overweight and obesity) (United Nations 2017).

### 2.2 The Intervention and How It Might Work

Breakfast consumption is of pivotal nutritional value during adolescence, a life stage characterized by rapid growth and development (Norris et al. 2022). Regular breakfast consumption is associated with better academic performance (Adolphus et al. 2013), improved overall diet quality (Giménez‐Legarre et al. 2020), and a reduced risk of being overweight (Ardeshirlarijani et al. 2019). What constitutes a breakfast meal and, thereby, what constitutes skipping the breakfast meal can vary significantly across countries and cultures. There is no standardized definition for breakfast habits in the scientific literature; thus, how authors define the concept of breakfast skipping varies across studies (Gibney et al. 2018). Factors such as lack of time, appetite, body weight control and socioeconomic status are associated with adolescents' breakfast habits (Esquius et al. 2021). A systematic review of 227 articles reported that 40% of sampled girls in LMICs skip breakfast (Keats et al. 2018). Breakfast skipping is associated with anemia (Andiarna 2018) and odds of anxiety, depression, and psychological distress (Zahedi et al. 2022).

### 2.3 Why It Is Important to Do This Review

Health and nutrition research is often not conducted in adolescent‐specific populations in LMICs, and little comparable information exists on dietary global trends specific to LMICs where malnutrition is prevalent. To the best of our knowledge, the evidence on the breakfast habits of adolescents in LMICs and their relationship with anthropometric, adiposity, and nutrition outcomes has yet to be systematically reviewed. This evidence will be critical to informing policy and programmatic adolescent‐specific decision‐making in LMICs.

## 3 Objectives

This review aims to examine the scientific evidence available from LMICs on the association between breakfast skipping and consumption habits and anthropometry‐ and nutrition‐related outcomes in adolescents 10–19 years old.

## 4 Methods

### 4.1 Criteria for Considering Studies for This Review

#### 4.1.1 Types of Studies

This systematic review and meta‐analysis were conducted in accordance with the Preferred Reporting Items for Systematic Reviews and Meta‐Analyses (PRISMA) guidelines (Page et al. 2021). The review protocol was registered with the International Prospective Register of Systematic Reviews (PROSPERO: CRD42023442910) and has previously been published (Fischer et al. 2024). Primary studies of the following study designs were eligible for inclusion: randomized controlled trials (inclusive of cluster randomized and crossover designs), non‐randomized intervention trials, and observational studies of prospective or retrospective cohort, cross‐sectional or case‐control. Reviews were not eligible for inclusion.

#### 4.1.2 Types of Participants

The target population was males and females aged 10*–*19 years, according to the World Health Organization's (WHO) definition of adolescence (World Health Organization n.d.). Studies that did not specifically recruit within the 10–19‐year age range but had overlapping age ranges were also considered, provided they had disaggregated adolescent data. Articles were eligible for inclusion if the mean or median age fell within the WHO‐defined age range.

#### 4.1.3 Types of Interventions

Studies that compared breakfast skipping and consumption habits in males and females aged 10*–*19 years were included. Given the various definitions possible for breakfast skipping and consumption across studies, any author‐defined instance of breakfast skipping/consumption frequency was included; however, authors generally defined skipping as not consuming breakfast at all or up to a maximum of two times per week. Studies were excluded if they only collected point prevalence data (i.e., breakfast on the day of the questionnaire: yes or no), as this did not provide information on the frequency of habitual breakfast behaviors among adolescents. Studies were excluded if they only assessed lunch, dinner, or snack consumption, as defined by study authors, skipped any other meal besides or in addition to breakfast, did not include a breakfast consumer control group, or co‐interventions were inconsistent across breakfast habit groups.

#### 4.1.4 Types of Outcome Measures

##### 4.1.4.1 Primary Outcomes

Our primary anthropometric/adiposity outcomes of interest were BMI, as a continuous variable, defined as weight in kilograms (kg) divided by height in meters squared), and categorical BMI, defined as overweight [BMI 25 to < 30], obese [BMI > 30], or overweight/obese [BMI > 25] according to the Centers for Disease Control and Prevention (CDC 2023), and BMI‐for‐age (*z*‐scores), defined as overweight > +1 SD and obesity > +2 SD, according to the WHO (Growth Reference 5–19 Years–BMI‐for‐Age (5–19 Years) n.d.). Our primary nutritional outcome of interest was the frequency (%) of anemia, defined as hemoglobin (Hb) < 115 g/L (11.5 g/dL or 7.14 mmol/L) for boys and girls 10–11 years old, Hb < 120 g/L (12.0 g/dL or 7.4 mmol/L) for boys 12–14 years old and girls 12–19 years old, and Hb < 130 g/L (13.0 g/dL or 7.7 mmol/L) for boys 15–19 years old, as per WHO guidelines (World Health Organization 2011).

##### 4.1.4.2 Secondary Outcomes

Secondary outcomes related to anthropometry/adiposity included waist circumference, waist‐to‐height ratio (WtHr), waist‐to‐hip ratio, height‐for‐age (stunting, *z*‐scores), skinfold thickness, hip circumference, mid‐upper arm circumference (MUAC) (adolescents 10–14 years) and bioelectric impedance. Secondary nutritional‐related outcomes were selected based on the prevalent micronutrient deficiencies commonly experienced by adolescents (Akseer et al. 2017; Society for Adolescent Health and Medicine 2018), which included frequency (%) of iron deficiency (defined as serum ferritin < 15 µg/L, as per WHO guidelines) (World Health Organization 2020), and iron deficiency anemia (relevant sub‐population Hb cut‐off + serum ferritin < 15 µg/L), iron levels as assessed by ferritin or soluble transferrin receptor, vitamin A levels as serum/plasma retinol or retinol‐binding protein concentrations, iodine levels as urinary iodine, zinc levels as plasma zinc, and calcium and vitamin D levels as 25(OH)D, parathyroid hormone or total calcium in serum/plasma or urine. Included studies reported on at least one of the primary or secondary outcomes.

##### 4.1.4.3 Duration of Follow‐Up

There was no minimum duration of follow‐up.

##### 4.1.4.4 Types of Settings

Any setting in LMICs, as defined by the World Bank (World Bank Country and Lending Groups–World Bank Data Help Desk n.d.).

### 4.2 Search Methods for Identification of Studies

#### 4.2.1 Electronic Searches

A detailed literature search strategy was developed in collaboration with a Public Health Informationist. We retrieved records from the following databases: MEDLINE via PubMed, EMBASE via Ovid, CINAHL, CENTRAL, and Web of Science. Our search strategy for MEDLINE PubMed can be found in Table S1 (online Supporting Information).

#### 4.2.2 Searching Other Resources

A gray literature search was conducted, which involved reviewing ongoing trials registered at ClinicalTrials.gov and the reference lists of published studies. The search was conducted from database inception through to June 28, 2023.

### 4.3 Data Collection and Analysis

#### 4.3.1 Description of Methods Used in Primary Research

#### 4.3.2 Selection of Studies

All the articles were uploaded into Covidence, and duplicates were automatically removed. In the first phase, two review authors (J.A.J.F. and J.T.) independently screened each relevant title and abstract to exclude irrelevant articles based on inclusion and exclusion criteria (Covidence n.d.). In the second phase, full texts of only those articles were retrieved, and the same two authors independently conducted a full‐text review to determine eligibility. At both stages, any discrepancies between reviewers were referred to the senior author (V.G.L.) and resolved through team discussion. Reasons for study exclusion were recorded at the full‐text level.

#### 4.3.3 Data Extraction and Management

Two authors (J.A.J.F. and J.T.) independently extracted data on each study's design, setting, population, intervention, comparator, outcomes, and measures of association into a form designed specifically for the review. Extracted data was then cross‐checked and merged. The extraction form was piloted with three studies to ensure clarity and comprehensiveness, and necessary changes were made based on the feedback of other study authors (D.I. and V.G.L.). For prospective studies, baseline data was extracted for the time point with the most available data. When information regarding any of the studies was unclear or unavailable, the corresponding authors of the original reports were contacted for further details.

#### 4.3.4 Assessment of Risk of Bias in Included Studies

Following data extraction, two reviewers (J.A.J.F. and J.T.) critically appraised the included studies. Since all included studies were of observational cohort and cross‐sectional design, the risk of bias within each study was assessed using the National Heart, Lung and Blood Institute's (NHLBI) Quality Assessment Tool for Observational Cohort and Cross‐Sectional Studies (NHLBI and NIH n.d.). One additional column concerning attrition rate, deemed important by the study team, was also added to the assessment process. Each study received an overall quality rating of “good,” “fair,” or “poor.”

#### 4.3.5 Measures of Treatment Effect

For continuous outcomes, we used standardized mean differences (SMDs) with 95% confidence intervals (95% CI) using Hedges' *g* method, and for dichotomous outcomes, we used odds ratios (ORs) with 95% CIs. Adjusted estimates were preferred for meta‐analyses. For observational studies reporting outcome numbers in each group instead of OR, we calculated the OR and standard error (SE), while for continuous outcomes, we used the mean (SD) and sample size reported for each group.

#### 4.3.6 Unit of Analysis Issues

Each study's reference and comparison groups were grouped according to the frequency of breakfast‐consuming/skipping groups to create single pairwise comparisons. The grouping included “regular” (eating breakfast 5–7 days per week), “infrequent” (eating breakfast 3–4 days per week), and “irregular” breakfast consumers (eating breakfast 0–2 days per week); and daily versus non‐daily breakfast consumption (grouping study author definitions including eating breakfast yes/no and eating breakfast regularly [6–7 days/week] yes/no). If there were four or more groups, the middle‐frequency groups were combined into a single “infrequent” consumption group. Meta‐analyses were conducted comparing regular versus infrequent, regular versus irregular, regular versus infrequent and irregular pooled, and daily versus non‐daily.

#### 4.3.7 Criteria for Determination of Independent Findings

In the case of multiple participant cohorts in one study, each cohort was treated as a separate study, contributing a single effect size estimate to the meta‐analysis, as long as the groups did not share a control group.

#### 4.3.8 Dealing With Missing Data

We contacted the corresponding study author when data was not readily available or calculable. Any missing data was assessed as part of the risk of bias.

#### 4.3.9 Assessment of Heterogeneity

We assessed statistical heterogeneity using the *χ* ^2^ test (*p* < 0.10) and *I* ^2^ statistic (*I* ^2^ > 50%). To ensure the robustness of the results, we conducted sensitivity analyses after removing studies with poor quality.

#### 4.3.10 Assessment of Reporting Biases

Publication bias was assessed through visual inspection of funnel plots for meta‐analysis with more than 10 studies.

#### 4.3.11 Data Synthesis

Using the generic inverse variance method of the R package “meta,” which requires estimates and SEs, random‐effects meta‐analysis models were used to pool data for each outcome measure from the included studies (Balduzzi et al. 2019). We pooled data reported by two or more studies, with the breakfast consumer group as the reference group. Statistical analyses were performed using RStudio, R version 4.2.2 (Rstudio Team 2022). *p* < 0.05 was considered statistically significant.

For some studies, results were reported separately for males and females, but when estimates were not available, raw numbers were used to calculate the estimate for the total group to be included in the meta‐analysis. For studies where meta‐analysis was not feasible or appropriate, we narratively synthesized the data following the Synthesis Without Meta‐Analysis (SWiM) reporting guidelines (BMJ 2020).

#### 4.3.12 Subgroup Analysis and Investigation of Heterogeneity

When outcomes had a sufficient number of studies, we conducted a gender subgroup analysis.

#### 4.3.13 Sensitivity Analysis

To explore high heterogeneity, we conducted sensitivity analyses in meta‐analyses with more than 10 studies, restricting them to include studies with only a fair quality rating (removing poor quality studies) based on the NHLBI Quality Assessment Tool. Pooled estimates for studies of different quality were presented separately in forest plots (fair vs. poor quality studies) and with the overall pooled estimate.

#### 4.3.14 Summary of Findings and Assessment of the Certainty of the Evidence

Quality appraisal was incorporated into the interpretation of results. The certainty of the evidence for each outcome was graded (J.A.J.F.) using the Grading of Recommendations Assessment, Development, and Evaluation (GRADE) approach (Guyatt et al. 2011). Based on the GRADE evaluation, the overall quality of the body of evidence was graded as “high,” “moderate,” “low,” or “very low” by outcome based on the GRADE domains (risk of bias, inconsistency, imprecision, indirectness, or publication bias). Evidence was downgraded or upgraded with clear reasoning based on this grading. All studies were initially considered as low evidence due to the observational study design (Guyatt et al. 2011). A summary of table findings was created using GRADEpro GDT, which informed our confidence in the effect estimates.

## 5 Results

### 5.1 Description of Studies

#### 5.1.1 Results of the Search

**Figure 1:**
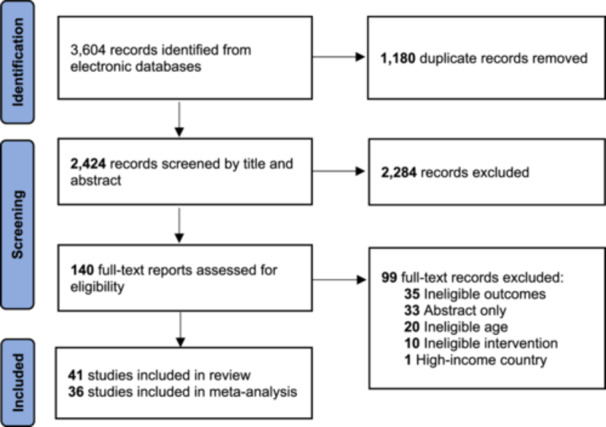
PRISMA flow diagram of study selection.

There were 3604 articles and abstracts initially identified from the literature search. After removing 1180 duplicates, 2424 unique records were screened for eligibility at the title and abstract level, where 2284 were excluded. Reviewers screened 140 full‐text articles, and 99 articles were excluded based on eligibility criteria. Thus, 41 studies were included in the systematic review, of which 36 were included in the meta‐analyses. Five studies were narratively included in the review but did not contribute data to the meta‐analyses as data was not in a form that could not be pooled with other studies or there was an insufficient number of studies to pool for meta‐analysis. Two studies, minimum, were required to conduct a meta‐analysis for a given outcome. A PRISMA Flow Diagram (Figure 1) depicts the search flow for this systematic review and reasons for study exclusion at the full‐text level.

#### 5.1.2 Included Studies

In total, there were 41 studies (160,708 individuals total), including 39 eligible cross‐sectional studies (158,548 adolescents) and 2 eligible prospective cohort studies (2160 adolescents) (Hassan et al. 2018; Mustafa et al. 2019). We did not identify any randomized or non‐randomized intervention trials. Characteristics of the eligible studies are presented in Table 1 by study design and alphabetical order by study author's last name. The sample size of the eligible studies ranged from 90 (Jain et al. 2020) to 36,956 (de Souza et al. 2021) adolescents. Studies primarily took place at school but also at the household (*n* = 1) (Boricic et al. 2014) and community level (*n* = 2) (Nurul‐Fadhilah et al. 2013; Sedibe et al. 2018; Wiafe et al. 2020). Twenty‐nine studies assessed BMI as a categorical variable (Akoto et al. 2022; Arora et al. 2012; Boricic et al. 2014; Chen et al. 2022; Cuesta et al. 2018; De Cnop et al. 2018; de Souza et al. 2021; Debeila et al. 2021; Duncan et al. 2011; Dundar and Oz 2012; El‐Kassas and Ziade 2017; Faizi et al. 2014; Fiuza et al. 2017; Hassan et al. 2018; Hatami et al. 2014; Khan et al. 2019; Koca et al. 2017; Lai et al. 2022; Maitland et al. 2015; Rashidi et al. 2007; Ribeiro et al. 2021; Santo Rocha et al. 2023; Saikia et al. 2016; Sayed et al. 2014; Soo et al. 2011; Sun et al. 2020; Talat and El Shahat 2016; Thompson‐McCormick et al. 2010; Xu et al. 2021), eight assessed BMI as a continuous variable (Ahadi et al. 2015; Cayres et al. 2015; Jain et al. 2020; Koca et al. 2017; Mustafa et al. 2019; Nurul‐Fadhilah et al. 2013; Shafiee et al. 2013; Silva et al. 2018), four assessed BMI‐for‐age (Faizi et al. 2014; Nurul‐Fadhilah et al. 2013; Sedibe et al. 2018; Tee et al. 2018), seven assessed waist circumference (Ahadi et al. 2015; de Souza et al. 2021; El‐Kassas and Ziade 2017; Mustafa et al. 2019; Nurul‐Fadhilah et al. 2013; Shafiee et al. 2013; Silva et al. 2018), seven assessed WtHr (Ahadi et al. 2015; De Cnop et al. 2018; de Souza et al. 2021; El‐Kassas and Ziade 2017; Nurul‐Fadhilah et al. 2013; Ribeiro et al. 2021; Shafiee et al. 2013), two assessed hemoglobin concentration (Ayogu et al. 2015; Jain et al. 2020), two assessed anemia (Jalambo et al. 2018; Sirajuddin and Masni 2015), and one assessed iron deficiency (Jalambo et al. 2018). Studies were conducted in Brazil (Cayres et al. 2015; De Cnop et al. 2018; de Souza et al. 2021; Duncan et al. 2011; Fiuza et al. 2017; Hassan et al. 2018; Norris et al. 2022; Ribeiro et al. 2021; Silva et al. 2018), Argentina (Cuesta et al. 2018), Turks and Caicos (Maitland et al. 2015), Serbia (Boricic et al. 2014), Turkey (Dundar and Oz 2012; Koca et al. 2017), Lebanon (El‐Kassas and Ziade 2017), Palestine (Jalambo et al. 2018), Iran (Ahadi et al. 2015; Hatami et al. 2014; Rashidi et al. 2007; Shafiee et al. 2013), India (Arora et al. 2012; Faizi et al. 2014; Jain et al. 2020; Saikia et al. 2016), Bangladesh (Khan et al. 2019), Indonesia (Sirajuddin and Masni 2015), China (Chen et al. 2022; Sun et al. 2020; Xu et al. 2021), Malaysia (Lai et al. 2022; Mustafa et al. 2019; Nurul‐Fadhilah et al. 2013; Soo et al. 2011; Tee et al. 2018), Fiji (Thompson‐McCormick et al. 2010), Algeria (Sayed et al. 2014), Egypt (Talat and El Shahat 2016), Ghana (Akoto et al. 2022), Nigeria (Ayogu et al. 2015), and South Africa (Debeila et al. 2021; Sedibe et al. 2018) and were published between 2007 and 2022.

**Table 1:** Characteristics of included studies in the systematic review.

Study ID	Country	Setting	Population	Age range	Sex	Total *n*	Age^a^	Breakfast habit classification^b^ (days/week)	Assessment method of breakfast skipping	Anthropometric outcomes assessed	Nutritional outcomes assessed
*Prospective cohort studies*											
Hassan et al. (2018)	Brazil	School	Twenty‐six 6th grade classes from four private and two public schools in Rio de Janeiro	10–16	54% boys; 46% girls	809	11.83 (1.15)	Regular (≥ 5) Irregular (≤ 4)	Three‐month qualitative FFQ adapted from a FFQ validated for Brazilian adolescents	Categorical BMI	
Mustafa et al. (2019)	Malaysia	School	Fifteen public secondary schools from Kuala Lumpur, Selangor, and Perak	13	37% boys; 63% girls	1351	12.9 (0.3)	Regular (7) Infrequent (1–6) Irregular (0)	Seven‐day diet history conducted via open‐ended interviews by dietitians	BMI Waist Circumference	
*Cross‐sectional studies*											
Ahadi et al. (2015)	Iran	School	Rural and urban elementary, intermediate, and high schools across 31 provinces	6–18	51% boys; 49% girls	13,486	12.4 (95% CI: 12.2–12.6)	Regular (5–7) Infrequent (3–4) Irregular (0–2)	Self‐administered questionnaire	BMI Waist‐to‐Height‐ratio Waist Circumference	
Akoto et al. (2022)	Ghana	School	Senior high schools in the Ho Municipality	13–19	15% boys; 85% girls	272	—	Daily Non‐daily	Repeated 24‐h dietary recall on two weekdays and one weekend administered via trained nutrition and dietetic master's students‐led interviews	Categorical BMI	
Arora et al. (2012)	India	School	8th (55%) and 10th (45%) grade in eight schools in Delhi	12–18	60% boys; 40% girls	2339	14.29	Regular (7) Infrequent (3–6) Irregular (0–2)	Self‐administered questionnaire adapted from instruments validated in adolescents	Categorical BMI	
Ayogu et al. (2015)	Nigeria	School	Schools in Ede‐Oballa, Nsukka	6–15	48% boys; 52% girls	450	33% 6–9 years; 26% 10–12 years; 41% 13–15 years	Regular (7) Infrequent (occasionally) Infrequent (4–5) Infrequent (1–3) Irregular (0)	Validated and pretested questionnaire administered via interviewing the parent‐participant pairs		Hemoglobin
Boričić et al. (2014)	Serbia	Household	Vojvodina, Belgrade, Western and Central and Southeastern Serbia	10–19	50% boys; 50% girls	2139	41% 10–13 years; 22% 14–15 years; 37% 16–19 years	Regular (7) Infrequent (1–6) Irregular (0)	Questionnaire administered face‐to‐face by trained interviewers in the home	Categorical BMI	
Cayres et al. (2015)	Brazil	School	Three large public primary schools in Presidente Prudente city	11–14	52% boys; 48% girls	120	11.7 (0.8)	Regular (7) Irregular (< 6)	Face‐to‐face interviews	BMI	
Chen et al. (2022)	China	School	Thirteen cities in Jiangsu Province	8–17	52% boys; 48% girls	36,849	56.9% 13–17 years	Daily Non‐daily AND Regular (7) Infrequent (1–6) Irregular (0)	Self‐administered questionnaire	Categorical BMI	
Cuesta et al. (2018)	Argentina	School	Twenty‐two schools in the District of General Pueyrredón	6–14	48% boys; 52% girls	1296	70% 9–14 years	Regular (> 4) Infrequent (< 4)	Questionnaire completed by parents under trained staff supervision	Categorical BMI	
Debeila et al. (2021)	South Africa	School	Four high schools in rural Fetakgomo Municipality	13–20	48% boys; 62% girls	378	16 (2)	Daily Non‐daily	Seven‐day validated FFQ	Categorical BMI	
De Cnop et al. (2018)	Brazil	School	Public (50%) and private (50%) elementary and high schools in Rio de Janeiro	10–19	50% boys; 50% girls	790	15.3 (0.9)	Daily Non‐daily	Self‐administered questionnaire	Categorical BMI Elevated Waist‐to‐Height‐ratio	
de Souza et al. (2021)	Brazil	School	Public and private schools in 5 Brazilian cities with > 100,000 inhabitants	12–17	50% boys; 50% girls	36,956	47% 12–14 years; 53% 15–17 years	Regular (7), Infrequent (1–6) Irregular (0)	Self‐administered questionnaire applied using an electronic data collector, a Personal Digital Assistant	Categorical BMI Elevated Waist Circumference Elevated Waist‐to‐Height‐ratio	
Duncan et al. (2011)	Brazil	School	Twenty‐two schools in São Paulo	7–18	47% boys; 53% girls	3397	B: 13.3 (2.6); G: 13.5 (2.7)	Regular (5–7) Infrequent (3–5) Irregular (0–2)	Self‐administered questionnaire	Categorical BMI	
Dundar and Oz (2012)	Turkey	School	Twenty secondary schools in Samsun	11–14	51% boys; 49% girls	2477	12.8 (0.9)	Regular Irregular	Self‐administered questionnaire under trained nurse supervision	Categorical BMI	
El‐Kassas and Ziade (2017)	Lebanon	School	7th–9th grade in public (54%) and private schools (46%) in Tripoli city	11–16	32% boys; 68% girls	311	Private schools: 13.28 (0.93); Public: 13.96 (1.3)	Regular Irregular	Questionnaire administered face‐to‐face by trained researchers	Categorical BMI Waist Circumference Waist‐to‐Height‐ratio	
Faizi et al. (2014)	India	School	Three schools in Aligarh city with Aligarh Muslim University Board of Examination affiliation	13–15	50% boys; 50% girls	1416	—	Regular (6–7) Infrequent (3–5) Irregular (0–2)	Pilot‐tested and validated questionnaire	BMI‐for‐age Categorical BMI	
Fiuza et al. (2017)	Brazil	School	Public and private schools in Cuiabá city	10–17	51% boys; 49% girls	1716	B: 57% 10–11 years; G: 55% 10–11 years	Daily breakfast consumption: yes or no	Self‐administered questionnaire by participant or parent/guardian	Categorical BMI	
Hatami et al. (2014)	Iran	School	Fifteen boys' schools and 15 girls' schools in Tehran	10–18	—	1157	—	Regular (4–7) Irregular (0–3)	Self‐administered questionnaire	Categorical BMI	
Jain et al. (2020)	India	School	Girls from urban (50%) and rural (50%) government schools of Ludhiana District	16–18	100% girls	90	17.2 (0.81)	Regular (7), Infrequent (2–3) Irregular (0)	Pre‐tested and validated qualitative FFQ	BMI	Hemoglobin
Jalambo et al. (2018)	Palestine	School	Five girls' secondary schools in Gaza	15–19	100% girls	330	16.74 (0.86)	Regular (7), Infrequent (1–6), Irregular (0)	12‐month self‐administered FFQ from the National Health Institute		Anemia Iron Deficiency
Khan et al. (2019)	Bangladesh	School	Eight secondary schools in Dhaka	12–17	50% boys; 50% girls	1476	14.23 (1.15)	Regular (5–7) Irregular (0–4)	Self‐administered questionnaire	Categorical BMI	
Koca et al. (2017)	Turkey	School	Ten schools in Isparta region	6–18	52% boys; 48% girls	7116	12.0 (4.58)	Regular (7) Infrequent (1–3) Irregular (0)	Self‐administered semi‐quantitative FFQ	Categorical BMI	
Lai et al. (2022)	Malaysia	School	From 1, 2, and 4 from eight government secondary schools in Seremban	12–18	46% boys; 54% girls	2659	14.19 (1.28)	Regular (7) Infrequent (1–6) Irregular (0)	Self‐administered questionnaire adapted from Malaysia's Adolescent Nutrition Survey 2017 with assistance given by researchers	Categorical BMI	
Maitland et al. (2015)	Turks and Caicos Islands	School	5th and 6th graders from six public elementary schools on Grand Turk, Providenciales, and South Caicos islands	—	45% boys; 55% girls	297	10.91 (1.01)	Daily Non‐daily	24‐h FFQ (Tuesday–Friday), read aloud in class and participants completed questionnaire	Categorical BMI	
Nurul‐Fadhilah et al. (2013)	Malaysia	Community	Kota Bharu city	12–19	44% boys; 56% girls	236	15.4 (1.9)	Regular (5–7) Irregular (0–5)	Pre‐tested self‐administered open‐ended questionnaire	BMI BMI‐for‐age Waist Circumference Waist‐to‐Height‐ratio	
Rashidi et al. (2007)	Iran	School	All educational zones in Tehran	11–16	46% boys; 54% girls	2321	B: 13.7 (1.54); G: 13.4 (1.64)	Regular (5–7) Infrequent (2–4) Irregular (0–1)	Data collected by trained nutritionists	Categorical BMI	
Ribeiro et al. (2021)	Brazil	School	State high schools in Campina Grande city	15–19	33% boys; 67% girls	571	60% 15–17 years; 40% 18–19 years	Regular (5–7) Irregular (0–4)	Question adopted from the *Pesquisa Nacional de* *Saúde do Escolar*	Categorical BMI Waist‐to‐Height‐ratio	
Santo Rocha et al. (2023)	Brazil	School	6th–9th grades in public and private schools	11–19	51% boys; 49% girls	16,554	14.1 (95% CI: 14.6, 14.2)	Regular (5–7) Irregular (0–4)	Questionnaire self‐administered via a study smartphone	Categorical BMI	
Saikia et al. (2016)	India	School	Schools of Dibrugarh town	10–14	54% boys; 46% girls	800	12 (1.33)	Regular (7) Infrequent (4–6) Irregular (0–3)	FFQ	Categorical BMI	
Sayed et al. (2014)	Algeria	School	4th–5th grades in public primary schools in Constantine district	10–11	—	399	10.54 (0.49)	Regular (7) Infrequent (1–6) Irregular (0)	Pretested parental questionnaire	Categorical BMI	
Sedibe et al. (2018)	South Africa	Community	Rural Agincourt and rural Soweto‐Johannesburg	11–13	49% boys; 51% girls	1768	13	Weekday breakfast consumption (excluding two weekend days): Regular (3–5) Irregular (0–2)	Questionnaire administered face‐to‐face by trained researchers	BMI‐for‐age	
Shafiee et al. (2013)	Iran	School	Urban and rural areas of 27 provinces	10–18	—	5604	14.7 (2.4)	Regular (6–7) Infrequent (3–5) Irregular (0–2)	Pretested parental questionnaire	BMI Waist Circumference Waist‐to‐Height‐ratio	
Silva et al. (2018)	Brazil	School	State‐funded schools in Juiz de Fora city	10–14	48% boys; 52% girls	493	10.82 (2.14)	Regular (7) Infrequent (1–6) Irregular (0)	Questionnaire administered face‐to‐face to parents and participants by trained researchers	BMI Waist Circumference	
Sirajuddin and Masni (2015)	Indonesia	School	Public elementary schools in Cambaya town	8–13	51% boys; 49% girls	120	29% 8–9 years; 60% 10–11 years; 11% 12–13 years	Regular (4–7) Infrequent (2–3)	Semi‐quantitative FFQ		Anemia
Soo et al. (2011)	Malaysia	School	Chinese primary school in Kota Bharu	10–12	52% boys; 48% girls	278	39% 10 years; 41% 11 years; 20% 12 years	Regular (7) Infrequent (1–6) Irregular (0)	Self‐administered 3‐day dietary record	Categorical BMI	
Sun et al. (2020)	China	School	4th–6th grade in 16 primary schools in Changsha city	7–15	53% boys; 47% girls	2185	10.83 (0.993)	Regular (7) Infrequent (1–6) Irregular (0)	Self‐administered questionnaire adapted from Malaysia's Adolescent Nutrition Survey 2017 with necessary assistance given by researchers	Categorical BMI	
Talat and El Shahat (2016)	Egypt	School	Government schools in Sharkia Governorate	12–15	48% boys; 52% girls	900	—	Regular (frequent) Infrequent (sometimes) Irregular (0)	Self‐administered questionnaire	Categorical BMI	
Tee et al. (2018)	Malaysia	School	Urban and rural primary secondary	6–17	48% boys; 52% girls	8322	36% 6–9 years; 28% 10–12 years; 28% 13–15 years; 8% 16–17 years;	Regular (5–7) Infrequent (3–4) Irregular (0–2)	Breakfast habits (defined as first eating after overnight sleep until 10 a.m. on weekdays or 11 a.m. on weekends) questionnaire administered via trained fieldworkers	BMI‐for‐age	
Thompson‐McCormick et al. (2010)	Fiji	School	Twelve secondary schools in one administrative sector	15–20	100% girls	523	16.68 (1.09)	Daily Non‐daily	Self‐administered questionnaire	Categorical BMI	
Xu et al. (2021)	China	School	Twelve schools in Shanghai, Fujian, Sichuan, and Shanxi provinces	16–18	50% boys; 50% girls	1200	33.3% 10 years; 33.3% 11 years; 33.3% 12 years	Regular (4–7) Irregular (0–3)	Self‐administered questionnaire under staff supervision according to the Chinese National Nutrition and Health Survey	Categorical BMI	

^a^ Mean (SD), mean (95% CI), % age range.

^b^ Categorical groupings of breakfast skipping by number of days per week consumption.

The definitions of breakfast skipping varied widely. Most studies defined the habit by weekly consumption frequency, with 13 studies comparing two frequency groups (Cayres et al. 2015; Cuesta et al. 2018; De Cnop et al. 2018; Hassan et al. 2018; Hatami et al. 2014; Khan et al. 2019; Nurul‐Fadhilah et al. 2013; Ribeiro et al. 2021; Santo Rocha et al. 2023; Sedibe et al. 2018; Sirajuddin and Masni 2015; Thompson‐McCormick et al. 2010; Xu et al. 2021) and 20 studies having multiple breakfast frequency groups (3–4 groups) (Ahadi et al. 2015; Arora et al. 2012; Ayogu et al. 2015; Boricic et al. 2014; de Souza et al. 2021; Duncan et al. 2011; Faizi et al. 2014; Jain et al. 2020; Jalambo et al. 2018; Koca et al. 2017; Lai et al. 2022; Mustafa et al. 2019; Rashidi et al. 2007; Saikia et al. 2016; Sayed et al. 2014; Shafiee et al. 2013; Soo et al. 2011; Sun et al. 2020; Talat and El Shahat 2016; Tee et al. 2018). Further, seven studies classified breakfast consumption as a binary (yes/no) variable (Akoto et al. 2022; Debeila et al. 2021; Dundar and Oz 2012; El‐Kassas and Ziade 2017; Fiuza et al. 2017; Maitland et al. 2015; Silva et al. 2018). Binary comparisons were meta‐analyzed separately from the weekly consumption frequency studies. One study collected both binary and frequency breakfast habit data (Chen et al. 2022). Exposure variables were analyzed differently across studies; regarding dietary assessment methods, one study utilized the 24‐h repeated dietary recall method (Akoto et al. 2022), two used 3‐ or 7‐day dietary records (Mustafa et al. 2019; Nurul‐Fadhilah et al. 2013), eight used food frequency questionnaires (Debeila et al. 2021; Hassan et al. 2018; Jain et al. 2020; Jalambo et al. 2018; Koca et al. 2017; Maitland et al. 2015; Saikia et al. 2016; Sirajuddin and Masni 2015), and others did not report the methods used. Twenty‐one studies were self‐administered (Ahadi et al. 2015; Arora et al. 2012; Cuesta et al. 2018; De Cnop et al. 2018; de Souza et al. 2021; Debeila et al. 2021; Duncan et al. 2011; Fiuza et al. 2017; Hatami et al. 2014; Jalambo et al. 2018; Khan et al. 2019; Koca et al. 2017; Lai et al. 2022; Maitland et al. 2015; Nurul‐Fadhilah et al. 2013; Santo Rocha et al. 2023; Sirajuddin and Masni 2015; Soo et al. 2011; Talat and El Shahat 2016; Thompson‐McCormick et al. 2010; Xu et al. 2021), while dieticians or researcher‐led interviews were conducted for seven (Ayogu et al. 2015; Boricic et al. 2014; Cayres et al. 2015; Chen et al. 2022; El‐Kassas and Ziade 2017; Sedibe et al. 2018; Tee et al. 2018). Parents participated or solely completed the questionnaire in five studies (Ayogu et al. 2015; Fiuza et al. 2017; Sayed et al. 2014; Shafiee et al. 2013; Silva et al. 2018).

#### 5.1.3 Excluded Studies

A total of 2563 articles were excluded, with 1180 being removed automatically by Covidence as duplicates and another 2284 excluded at the first title and abstract screening phase. Ninety‐nine articles were excluded at the full‐text level for the reasons of ineligible outcomes (not a priori determined primary and secondary outcomes), only a study abstract, outside of the 10–19 adolescent age range, ineligible intervention, or taking place in a high‐income setting.

### 5.2 Risk of Bias in Included Studies

**Figure 2:**
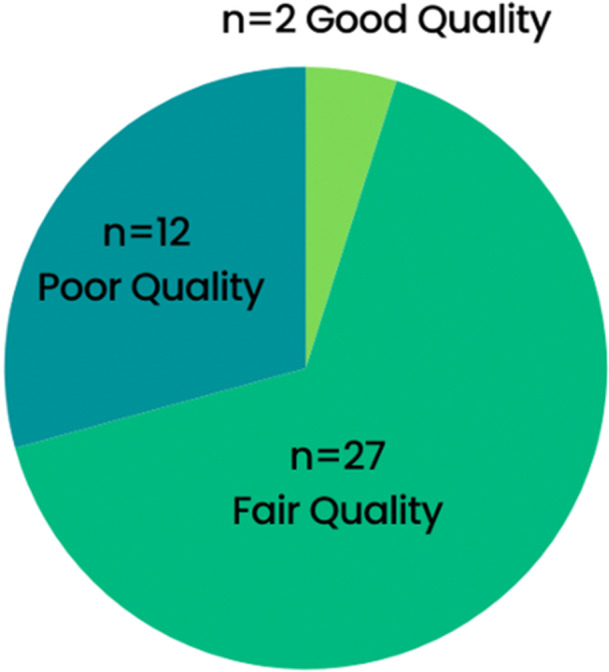
Overall quality rating of 41 included studies.

Table 2 provides a summary of the risk of bias domains in each study. The studies were judged to be of varying levels of quality, with 2 rated as good quality, 27 as fair quality, and 12 having an overall poor quality rating, as shown in Figure 2. The studies rated as poor quality commonly lacked sample size justification, a priori statistical analysis plan, outcome assessor blinding, and validated tools to assess exposure. Due to the cross‐sectional nature of the majority of studies, the exposure was not assessed before the outcome measure, nor was there a sufficient timeframe to see an effect. There was also some evidence suggesting publication bias, as detected by visual inspection of funnel plot asymmetry in Figures S1 and S2.

**Table 2:** Study quality assessment using the NHLBI Quality Assessment Tool for observational cohort and cross‐sectional studies.

Study ID	1. Clear research question	2. Clear study population	3. 50% participation rate	4. Groups recruited from the same population	5. Sample size justification	6. Exposure assessed before outcome measure	7. Sufficient timeframe to see effect	8. Different levels of exposure of interest	9. Exposure measures and assessment	10. Repeated exposure assessment	11. Outcome measures	12. Blinding of outcome assessors	13. Follow‐up rate	14. Statistical analysis	15. Overall quality rating
*Prospective cohort studies*															
Hassan et al. (2018)	Y	Y	Y	Y	N	N	N	Y	Y	Y	Y	N	Y	Y	Good
Mustafa et al. (2019)	Y	Y	U	Y	N	N	N	Y	Y	N	Y	N	U	Y	Fair
*Cross‐sectional studies*															
Ahadi et al. (2015)	Y	Y	Y	Y	Y	U	U	Y	Y	U	Y	N	U	Y	Good
Akoto et al. (2022)	Y	Y	U	Y	Y	U	U	N	U	N	Y	N	U	Y	Fair
Arora et al. (2012)	Y	Y	Y	Y	N	N	N	Y	Y	N	Y	N	U	Y	Fair
Ayogu et al. (2015)	Y	Y	U	Y	Y	N	N	N	N	N	Y	N	U	Y	Fair
Boričić et al. (2014)	Y	Y	Y	Y	N	N	N	Y	Y	N	Y	Y	U	Y	Fair
Cayres et al. (2015)	Y	Y	N	Y	Y	N	N	N	Y	N	Y	N	U	Y	Fair
Chen et al. (2022)	Y	Y	Y	Y	N	N	N	N	N	N	Y	N	U	Y	Poor
Cuesta et al. (2018)	Y	Y	Y	Y	Y	N	N	N	Y	N	Y	N	U	N	Fair
Debeila et al. (2021)	Y	Y	Y	Y	Y	N	N	N	N	N	Y	N	U	Y	Poor
De Cnop et al. (2018)	Y	Y	Y	Y	N	N	N	Y	Y	N	Y	N	U	Y	Fair
de Souza et al. (2020)	Y	Y	Y	Y	Y	N	N	Y	Y	N	Y	Y	U	Y	Fair
Duncan et al. (2011)	Y	Y	Y	Y	N	U	U	Y	Y	N	N	N	U	Y	Fair
Dundar and Oz (2012)	Y	Y	U	Y	N	N	N	N	N	N	N	N	U	N	Poor
El‐Kassas and Ziade (2017)	Y	Y	U	Y	Y	N	N	N	N	N	Y	N	U	N	Poor
Faizi et al. (2014)	Y	Y	U	Y	N	N	N	Y	Y	N	Y	N	U	N	Fair
Fiuza et al. (2017)	Y	Y	U	Y	N	N	N	Y	Y	N	Y	Y	U	Y	Fair
Hatami et al. (2014)	Y	Y	Y	Y	Y	N	N	N	N	N	Y	N	U	Y	Fair
Jain et al. (2020)	Y	Y	U	Y	N	N	N	Y	Y	N	Y	Y	U	N	Poor
Jalambo et al. (2018)	Y	Y	U	Y	Y	N	N	N	N	N	Y	N	U	Y	Poor
Khan et al. (2019)	Y	Y	Y	Y	N	N	N	N	N	N	Y	N	U	Y	Fair
Koca et al. (2017)	Y	Y	U	N	N	N	N	N	Y	N	Y	N	U	N	Poor
Lai et al. (2022)	Y	Y	U	Y	Y	N	N	N	Y	N	Y	N	U	U	Fair
Maitland et al. (2015)	Y	Y	Y	Y	N	N	N	N	Y	N	Y	N	U	Y	Fair
Nurul‐Fadhilah et al. (2013)	Y	Y	U	Y	Y	N	N	Y	Y	N	Y	N	U	Y	Fair
Rashidi et al. (2007)	Y	N	U	U	N	N	N	Y	Y	N	Y	N	U	U	Poor
Ribeiro et al. (2021)	Y	Y	N	Y	Y	N	N	N	Y	N	Y	N	U	U	Poor
Santo Rocha et al. (2023)	Y	Y	U	Y	N	N	N	N	Y	N	Y	N	U	Y	Fair
Saikia et al. (2016)	Y	Y	U	Y	Y	N	N	Y	N	N	Y	N	U	U	Poor
Sayed et al. (2014)	Y	Y	U	Y	N	N	N	Y	N	N	Y	N	U	N	Poor
Sedibe et al. (2018)	Y	Y	U	Y	N	N	N	Y	Y	N	Y	N	U	Y	Fair
Shafiee et al. (2013)	Y	Y	U	Y	N	N	N	Y	Y	N	Y	N	U	U	Fair
Silva et al. (2018)	Y	Y	U	Y	Y	N	N	N	Y	N	Y	N	U	Y	Fair
Sirajuddin and Masni (2015)	Y	Y	Y	Y	N	N	N	Y	Y	N	Y	N	U	N	Fair
Soo et al. (2011)	Y	Y	N	Y	Y	N	N	Y	Y	N	Y	N	U	U	Poor
Sun et al. (2020)	Y	Y	U	Y	Y	N	N	Y	Y	N	Y	N	U	Y	Fair
Talat and El Shahat (2016)	Y	Y	N	Y	Y	N	N	Y	Y	N	Y	N	U	Y	Fair
Tee et al. (2018)	Y	Y	N	Y	Y	N	N	Y	Y	N	Y	N	U	Y	Fair
Thompson‐McCormick et al. (2010)	Y	Y	U	Y	N	N	N	Y	Y	N	Y	N	U	Y	Fair
Xu et al. (2021)	Y	Y	U	Y	N	N	N	N	Y	N	Y	N	U	Y	Fair

### 5.3 Synthesis of Results

An article by Cayres et al. was excluded from the meta‐analysis on the basis that no common outcomes of interest were reported and could not be pooled with other studies (Cayres et al. 2015). Four studies presented outcome data that could not be pooled (Ayogu et al. 2015; Dundar and Oz 2012; Sedibe et al. 2018; Silva et al. 2018). Therefore, 36 studies were available for meta‐analysis.

#### 5.3.1 Primary Anthropometric Outcomes: BMI

**Figure 3:**
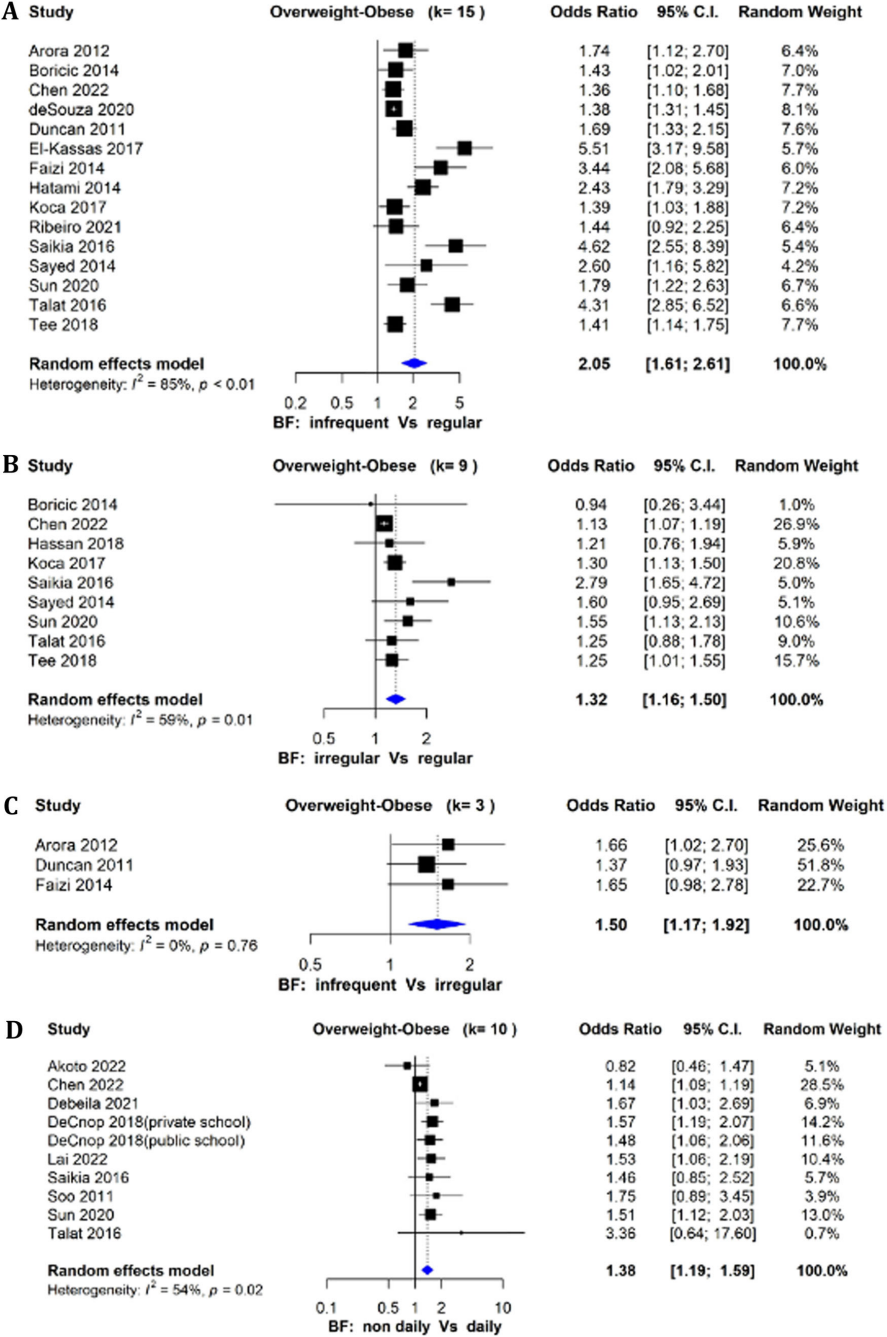
(A) Forest plot of studies (*n* = 15) comparing infrequent and regular breakfast consumption on odds of overweight/obesity in adolescents in LMICs (OR > 1 favors infrequent, < 1 favors regular). (B) Forest plot of studies (*n* = 9) comparing irregular and regular breakfast consumption on odds of overweight/obesity (OR > 1 favors irregular, < 1 favors regular). (C) Forest plot of studies (*n* = 3) comparing infrequent and irregular breakfast consumption on odds of overweight/obesity (OR > 1 favors infrequent, < 1 favors irregular). (D) Forest plot of studies (*n* = 10) comparing non‐daily and daily breakfast consumption on odds of overweight/obesity (OR > 1 favors non‐daily, < 1 favors daily).

Fifteen observational (cross‐sectional) studies investigated the relationship between skipping breakfast (infrequency vs. regular) and combined overweight/obesity (categorical BMI outcome). Adolescents who infrequently ate breakfast (0–2 days/week) were twice as likely to be overweight/obese (OR: 2.05, 95% CI: 1.61–2.61; *I* ^2^ = 85%; *n* = 15 studies) compared to regular breakfast consumers (5–7 days/week) (Figure 3A). Among nine observational studies, irregular breakfast consumers (3–4 days/week) had a 32% higher likelihood of being overweight/obese (OR: 1.32, 95% CI: 1.16–1.50; *I* ^2^ = 59%) compared to irregular breakfast consumers (Figure 3B). Those who irregularly ate breakfast had a 50% higher likelihood of being overweight/obese (OR: 1.50, 95% CI: 1.17–1.92; *I* ^2^ = 0%; *n* = 3 studies) compared to infrequent breakfast consumers (Figure 3C). The odds of non‐daily breakfast consumers being overweight/obese were 38% higher compared to daily consumers (OR: 1.38, 95% CI: 1.19–1.59; *I* ^2^ = 54%; *n* = 10 studies) (Figure 3D).

Significant heterogeneity was found among the studies comparing infrequent versus regular breakfast consumption on overweight/obesity, and evidence of publication bias (Egger's test *p* = 0.0083) was observed (Figure S1), with larger studies being more likely to be published. Moderate heterogeneity was found among studies comparing non‐daily versus daily breakfast consumption on overweight/obesity, with evidence of publication bias (Egger's test *p* = 0.0022) observed (Figure S2).

Some studies did not pool overweight and obesity but assessed the association between breakfast habits and being overweight and obese separately. The meta‐analysis of four studies (Ahadi et al. 2015; Cuesta et al. 2018; Rashidi et al. 2007; Talat and El Shahat 2016) found a 2.25 increased odds of infrequent breakfast consumers being overweight compared to regular breakfast eaters (95% CI: 1.02–4.95; *I* ^2^ = 97%) (Figure S3A). Adolescents who irregularly ate breakfast were more likely to be overweight (OR: 1.24, 95% CI: 1.05–1.48; *I* ^2^ = 0%; *n* = 3 studies) compared to regular breakfast consumers (Figure S3B) (Rashidi et al. 2007; Talat and El Shahat 2016; Zahedi et al. 2022). Further, there was a nonsignificant difference in odds of being overweight between pooled infrequent/irregular breakfast consumers and regular breakfast consumers (OR: 1.59, 95% CI: 0.95–2.65; *I* ^2^ = 96%; *n* = 3 studies) (Figure S3C) (Khan et al. 2019; Santo Rocha et al. 2023; Xu et al. 2021).

Four studies evaluated the odds of obesity between infrequent and regular breakfast consumers (Ahadi et al. 2015; Cuesta et al. 2018; Rashidi et al. 2007; Talat and El Shahat 2016). For infrequent breakfast consumers, there were 3.77 greater odds of obesity (95% CI: 1.95–7.31; *I* ^2^ = 95%) compared to regular consumers (Figure S3D). Pooled from four study populations (Fiuza et al. 2017; Maitland et al. 2015; Thompson‐McCormick et al. 2010), non‐daily breakfast consumers had 1.40 greater odds of obesity (95% CI: 1.05–1.87; *I* ^2^ = 72%) compared to daily breakfast consumers (Figure S3E). There were no significant differences in odds of being obese between irregular versus regular (OR: 1.39, 95% CI: 0.97–1.99; *I* ^2^ = 60%) nor non‐daily versus daily breakfast consumers (OR: 1.10, 95% CI: 0.99–1.23; *I* ^2^ = 0%).

**Figure 4:**
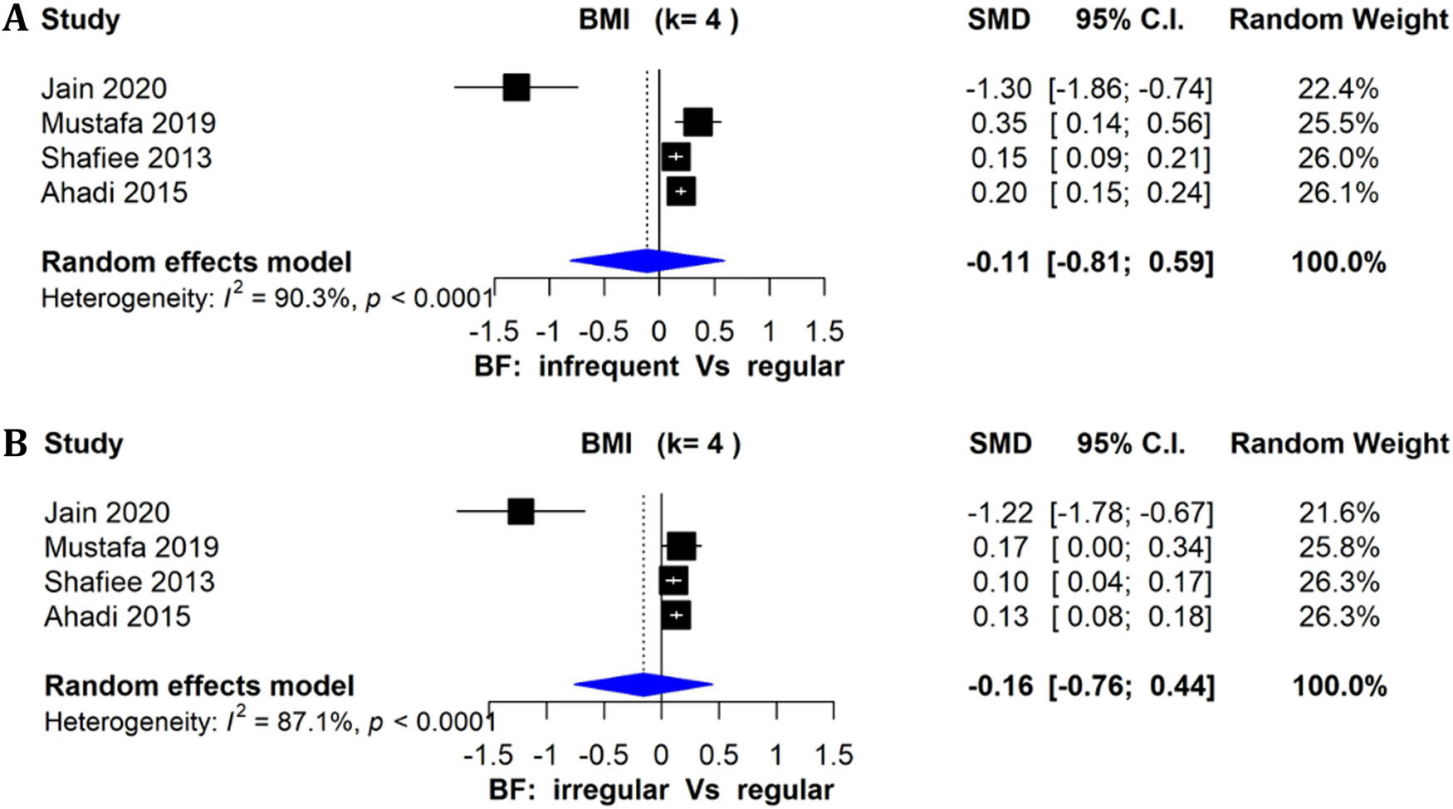
(A) Forest plot of studies (*n* = 4) comparing infrequent and regular breakfast consumption on BMI (SMD > 0 favors infrequent, < 0 favors regular). (B) Forest plot of studies (*n* = 4) comparing irregular and regular breakfast consumption on BMI (SMD > 0 favors irregular, < 0 favors regular).

BMI was also assessed in four studies as a continuous outcome (Ahadi et al. 2015; Jain et al. 2020; Mustafa et al. 2019; Shafiee et al. 2013), where pooled SMD (95% CI) by random‐effects model was not significantly different between adolescents consuming breakfast infrequently versus regularly (−0.11, [−0.81 to 0.59]; *I* ^2^ = 90.3%) (Figure 4A). Similarly, the pooled effect size for BMI (SMD and 95% CI) between irregular and regular consumers was −0.16 (−0.76, 0.44; *I* ^2^ = 87.1%; *n* = 4 studies), showing no significant differences (Figure 4B). For these two analyses, although the regular breakfast‐consuming group had a higher BMI in the Jain et al. study, 83% of the group was normal weight, whereas the infrequent and irregular groups were 63% and 67% underweight, respectively.

Cayres et al. reported no differences in mean (SD) BMI in those who never skipped breakfast than those who skipped breakfast at least 1 day per week (22.2 [3.8] vs. 20.6 [4.4], *p* = 0.104) (Cayres et al. 2015). Another study stated that the obesity prevalence in adolescents who skipped breakfast was higher than in their counterparts (*X* ^2^ = 9.1; *p* < 0.05) (Dundar and Oz 2012). In a report by Fiuza et al. the prevalence of breakfast skipping was associated with weight status, but after adjusting for age and socioeconomic status, it was only significantly associated with obesity for boys (prevalence ratio = 1.76 [95% CI: 1.28, 2.44], *p* = 0.01) (Fiuza et al. 2017). Duncan reported that the odds of overweight/obesity were 0.59 times lower (95% CI: 0.46–0.75, *p* < 0.01) in adolescents who ate breakfast on more than 5 days in the previous week than those who did not (Duncan et al. 2011). In a study by Thompson‐McCormick, no significant association between breakfast skipping and overweight/obesity was seen after adjusting for eating pathology (based on scores from an eating disorder examination questionnaire) (Thompson‐McCormick et al. 2010) Mustafa et al. revealed that each additional day of breakfast consumption per week was associated with a lower BMI (−0.34, [95% CI: −0.02, −0.66], *p* = 0.003) (Mustafa et al. 2019). Sedibe et al. reported irregular breakfast consumption was associated with an increased risk of overweight and obesity (measured by BMI‐for‐age *z*‐scores) in adolescents, with variations by age group and day of the week. Among early adolescents (Year 13, ages 10/13–14/15), irregular weekday breakfast consumption was associated with higher odds of being overweight or obese (OR = 1.38, 95% CI = 1.007–1.896, *p* ≤ 0.05). For mid‐adolescents (Year 15, ages 14/15–17), irregular weekend breakfast consumption showed a similar association with increased odds of overweight and obesity (OR: 1.53, 95% CI: 1.099–2.129, *p* ≤ 0.01) (Sedibe et al. 2018). Finally, Tee et al. reported mean (SD) BMI‐for‐age *z*‐scores to be significantly higher among adolescent breakfast skippers in both boys (0.5 [1.5], *p* = 0.043) and girls (0.3 [1.4], *p* = 0.05) (Tee et al. 2018).

#### 5.3.2 Primary Nutritional Outcomes: Anemia

**Figure 5:**
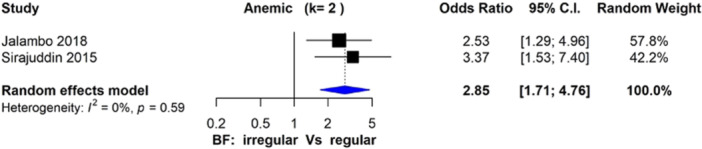
Forest plot of studies (*n* = 2) comparing irregular and regular breakfast consumption on odds of anemia in adolescents in LMICs (OR > 1 favors irregular, < 1 favors regular).

Two studies compared the associations of breakfast skipping with anemia prevalence in adolescents (Jalambo et al. 2018; Sirajuddin and Masni 2015). Adolescents who irregularly consumed breakfast were more than twice as likely to have anemia (OR: 2.85, 95% CI: 1.71, 4.76; *I* ^2^ = 0%; *n* = 2 studies) compared to regular breakfast consumers (Figure 5). There were insufficient studies (*n* = 1) that reported on breakfast skipping and hemoglobin concentration to conduct a meta‐analysis, yet Jain et al. revealed a significant (*p* < 0.05) difference in hemoglobin levels between regular breakfast consumers (12.04 ± 1.02 g/dL) in comparison to occasional and never breakfast consumers (10.46 ± 1.37 g/dL and 9.38 ± 1.34 g/dL, respectively) (Jain et al. 2020). Ayogu et al. reported that as the frequency of skipping breakfast increased, hemoglobin levels decreased (*b* = 1.542, *p* < 0.001) (Ayogu et al. 2015).

#### 5.3.3 Secondary Anthropometric Outcomes: Waist Circumference and WtHr

Three studies compared the associations of breakfast habits with the secondary waist circumference outcome as a continuous variable (Ahadi et al. 2015; Mustafa et al. 2019; Shafiee et al. 2013). There were no significant differences in waist circumference between adolescents who infrequently consume breakfast compared to those who regularly consume breakfast (Figure S4A) or when comparing irregular to regular breakfast consumers (Figure S4B). Three studies (de Souza et al. 2021; El‐Kassas and Ziade 2017; Ribeiro et al. 2021) evaluated waist circumference as a categorial variable, and the pooled OR (1.82, 95% CI: 0.97, 3.41; *I* ^2^ = 84%, *p* = 0.01) revealed significant odds of elevated waist circumference among infrequent breakfast consumers in comparison to those regularly taking breakfast (Figure S4C **)**. Silva et al. observed that skipping breakfast was not associated with increased waist circumference (prevalence ratio = 1.06 [95% CI: 0.56–2.01], *p* = 0.855). Further, Mustafa et al. reported that each additional day of breakfast consumption per week was associated with a lower waist circumference, but it was no longer significant after adjusting for BMI (Mustafa et al. 2019).

Three studies measured elevated WtHr (de Souza et al. 2021; El‐Kassas and Ziade 2017; Ribeiro et al. 2021), in which the pooled OR (95% CI) indicated a 1.67 odds of elevated WtHr (1.17–2.38; *I* ^2^ = 65%) amongst adolescent infrequent breakfast consumers than regular breakfast consumers (Figure S5A). Among two studies that collected continuous WtHr data, no SMD was observed between irregular compared to regular breakfast consumers (0.01, 95% CI: −0.03 to 0.05; *I* ^2^ = 0%) but there was a significant mean difference between infrequent compared to regular breakfast consumers (0.07, 95% CI: 0.03–0.10; *I* ^2^ = 0%) (Figure S5B,C). In another analysis of two populations comparing infrequent plus irregular breakfast consumers versus regular, a significant mean difference was observed (1.49, 95% CI: 0.51–2.47; *I* ^2^ = 90.8%), although limited by sample size and lacking clinical significance (Figure S5D).

#### 5.3.4 Secondary Nutritional Outcomes: Iron Deficiency

We only identified one study that collected data on the association between breakfast habits and iron deficiency (Jalambo et al. 2018); therefore, no summary statistic was produced. Jalambo et al. reported that Palestinian female adolescent infrequent and irregular breakfast consumers were more likely to be iron deficient compared to regular breakfast consumers (OR = 4.01, 95% CI: 1.17–9.33, *p* = 0.006 and OR = 3.10, 95% CI: 1.56–6.17, *p* = 0.003, respectively).

### 5.4 Sensitivity Analyses

To explore high heterogeneity, we conducted sensitivity analyses in meta‐analyses with more than 10 studies, restricting them to include studies with only a fair quality rating (removing poor quality studies) based on the NHLBI Quality Assessment Tool. With the removal of six studies of poor quality (Chen et al. 2022; El‐Kassas and Ziade 2017; Koca et al. 2017; Ribeiro et al. 2021; Saikia et al. 2016; Sayed et al. 2014), the summary OR (95%) among nine studies for infrequent breakfast consumption versus regular consumption was 1.94 (1.51–2.51; *I* ^2^ = 86%) for overweight and obesity, consistent with the primary analysis, yet heterogeneity remained high (Figure S6A). For non‐daily versus daily breakfast consumption, with the removal of four studies (Chen et al. 2022; Debeila et al. 2021; Saikia et al. 2016; Soo et al. 2011), the summary OR (95%) among seven studies was 1.50 (1.30–1.73; *I* ^2^ = 0%) for overweight and obesity, again consistent with the primary analysis (Figure S6B). To explore whether heterogeneity was driven by adjusted estimates, we also conducted the analysis using unadjusted estimates where available (except Jalambo et al. 2018; Sun et al. 2020; and Talat and El Shahat 2016), with minimal changes observed in pooled estimates, *p*‐values, and *I* ^2^ (data not shown).

### 5.5 Subgroup Analyses

A subgroup analysis of the association of breakfast habits on BMI in adolescent boys (Chen et al. 2022; Hassan et al. 2018; Tee et al. 2018) resulted in nonsignificant odds of overweight or obesity among irregular breakfast consumers in comparison to those who regularly ate breakfast (1.10, 95% CI: 1.01–1.19, *I* ^2^ = 0%) (Figure S7A). Similarly, no differences in odds were observed between infrequent and regular male adolescent breakfast consumers (1.23, 95% CI: 1.00–1.51; *I* ^2^ = 0%) (Figure S6B). Further, a subgroup analysis among adolescent girls (Chen et al. 2022; Hassan et al. 2018; Tee et al. 2018) revealed a 1.32 odds of being overweight or obese among irregular breakfast consumers in comparison to those regularly taking breakfast (95% CI: 1.08–1.62, *I* ^2^ = 71%) (Figure S7C). However, no differences in odds for the same outcome were observed for infrequent versus regular breakfast habits in adolescent girls (1.57 [95% CI: 1.20–2.06], *I* ^2^ = 25%) (Figure S7D).

## 6 Discussion

### 6.1 Summary of Main Results

Adolescence is recognized as a critical window for growth and development, and optimal nutrition and health are vital to long‐term health and setting girls up to thrive and for healthy reproductivity years to break the cycle of malnutrition. However, in LMICs, the health and nutrition of adolescents have often been neglected due to a lack of adolescent‐specific national data, funding indicators, and targets, such as the need to extend the current 50% anemia reduction target in 15–49‐year‐olds to early adolescents (10–14 years) and to boys (Natasha and Wrottesley 2023).

This review aimed to synthesize evidence on the relationship between breakfast frequency and anthropometric and nutritional outcomes in adolescents residing in LMICs. The findings of the current meta‐analysis demonstrated that breakfast skipping increased the odds of overweight/obesity in adolescents living in LMICs. Adolescents who ate breakfast infrequently had 2.05 odds of being overweight or obese compared to those who ate breakfast regularly. The OR was 1.32 for those who occasionally ate breakfast compared to those who regularly ate breakfast and 1.41 for those who did not eat breakfast daily compared to those who ate breakfast every day. Since most studies had cross‐sectional designs (only two cohort studies), we cannot determine the cause‐effect relationship between breakfast skipping and overweight/obesity. Establishing causal relationships between breakfast habits and health outcomes is challenging due to confounding factors such as physical activity, sleep patterns, or SES, which can independently influence health outcomes (Mitchell et al. 2013). Other challenges may include variability in breakfast composition, reliance on self‐reported data, and potential reverse causality (e.g., health conditions influencing breakfast habits). Findings should be interpreted cautiously due to the low‐quality evidence assessment. More trials are needed to elucidate this link, including an assessment of breakfast quantity and nutritional quality.

The rising prevalence of overweight and obesity among adolescents is a concerning trend globally, particularly in LMICs (Jebeile et al. 2022). In 57 LMICs, the prevalence of overweight or obesity among adolescents is 21.4% (95% CI: 18.6%, 24.2%) (Caleyachetty et al. 2018). The growing obesity epidemic has been driven by the global food system's transitions towards diets increasingly high in processed, energy‐dense, and micronutrient‐poor foods, an unhealthy lifestyle that adolescents can be highly susceptible to adopting, which may persist into adulthood. In LMICs, this may be exacerbated by rapid urbanization, resulting in this dietary intake pattern, food insecurity, and low physical activity levels (Ford et al. 2017). Overweight and obesity result from a complex interplay of factors, of which breakfast skipping may play a role, as demonstrated by this review and an extended body of evidence (Monzani et al. 2019). Several mechanisms may contribute to this observed relationship, including disruptions in circadian rhythms and the significance of nutrient timing. Circadian clocks regulate key biological processes, and breakfast consumption plays a role in maintaining their synchronization, thereby supporting metabolism and weight management (Gwin and Leidy 2018; Qin et al. 2003; Yoshizaki et al. 2013). Evidence from a randomized crossover study involving healthy individuals with type 2 diabetes indicates that eating breakfast helps preserve the normal oscillations of peripheral clock genes, whereas skipping breakfast disrupts these rhythms (Jakubowicz et al. 2017). Adolescent breakfast skippers have been shown to exhibit a compensatory increase in energy intake later in the day, particularly through snack foods, although the literature remains inconclusive (Barrett et al. 2018; Sjöberg et al. 2003).

### 6.2 Overall Completeness and Applicability of Evidence

The review followed best practice systematic review methodology, thoroughly searched numerous databases, evaluated study quality, and conducted sensitivity analyses. The adolescent study population was wide, representing a variety of food habits across six continents. However, the review acknowledges certain limitations. Firstly, most studies had a cross‐sectional design; consequently, any causal relationship remains to be determined. Between‐study heterogeneity was not markedly reduced following sensitivity analysis by study quality and unadjusted analyses. The high heterogeneity may be due to the variations in study design, populations, and breakfast definitions and may affect the reliability of the pooled findings. Additionally, some studies used unstandardized, non‐pretested, and unvalidated questionnaires to gather exposure variable data. Self‐reported data in 21 studies further limits questionnaire reliability. We did not collect data on breakfast timing and composition, which may play roles in adolescents' obesity risk. The reasons for skipping breakfast were also not evaluated, as these areas were beyond the scope of the present review. Educational level, ethnicity, physical activity level, parental guidance, age, and gender are potential factors that can impact breakfast skipping. Most studies adjusted for age and sex, but not all adjusted for relevant confounding factors known to be significantly positively associated with BMI (Lopez Barrera and Shively 2022). This lowered their quality assessment score and limited the findings of the present review. Although we searched numerous appropriate databases, PsycINFO was not searched but should be searched in future related reviews. Future qualitative research is needed to understand the causal factors informing breakfast skipping as well as the dietary data necessary to inform appropriate public health guidelines.

### 6.3 Quality of the Evidence

Tables 3, 4, 5, 6 summarize the pooled analyses of findings and the certainty of the evidence for anthropometric and nutritional outcomes when comparing adolescent breakfast consumers and skippers. The evidence certainty was either low or very low for all outcomes assessed in this systematic review. Each outcome started with a compulsory low rating because the data was from cross‐sectional or cohort studies, and the certainty of the evidence was further downgraded for certainty, mainly due to the risk of bias and inconsistency.

**Table 3:** GRADE of evidence assessment for key review findings for infrequent versus regular adolescent breakfast consumers.

Outcomes	No. of participants (Studies)	Certainty of the evidence (GRADE)	Reason for downgrading
BMI	20,531 (3 cross‐sectional studies, 1 cohort)	⨁◯◯◯ Very low	*I* ^2^ = 90.3%, 95% CI spans from [−0.81 to 0.59]; Mild publication bias detected through visual inspection of funnel plot.
BMI‐for‐age	3184 (3 cross‐sectional studies)	⨁⨁◯◯ Low	
Overweight/obese	104,857 (15 cross‐sectional studies)	⨁◯◯◯ Very low	33% of studies were assessed to have an overall poor‐quality rating.
Overweight	18,003 (4 cross‐sectional studies)	⨁◯◯◯ Very low	25% of studies were assessed to have an overall poor‐quality rating; *I* ^2^ = 97%, 95% CI spans from [1.02–4.95].
Obese	18,003 (4 cross‐sectional studies)	⨁◯◯◯ Very low	25% of studies were assessed to have an overall poor‐quality rating; *I* ^2^ = 95%, 95% CI spans from [1.05–7.31].
Waist circumference	20,441 (2 cross‐sectional studies, 1 cohort)	⨁⨁◯◯ Low	
Elevated waist‐to‐height ratio	37,665 (3 cross‐sectional studies)	⨁◯◯◯ Very low	66% of studies were assessed to have an overall poor‐quality rating.

Abbreviations: CI, confidence interval; GRADE, Grading of Recommendations Assessment, Development, and Evaluation; OR, odds ratio; SMD, standardized mean difference.

**Table 4:** GRADE of evidence assessment for key review findings for irregular versus regular adolescent breakfast consumers.

Outcomes	No. of participants (Studies)	Certainty of the evidence (GRADE)	Reason for downgrading
BMI	20,531 (3 cross‐sectional studies, 1 cohort)	⨁◯◯◯ Very low	25% of studies were assessed to have an overall poor‐quality rating; *I* ^2^ = 87.1%, 95% CI spans from [0.76, 0.44].
Overweight/obese	59,519 (8 cross‐sectional studies, 1 cohort)	⨁◯◯◯ Very low	33% of studies were assessed to have an overall poor‐quality rating.
Waist circumference	20,441 (2 cross‐sectional studies, 1 cohort)	⨁⨁◯◯ Low	
Waist‐to‐height ratio	19,090 (1 cross‐sectional study, 1 cohort)	⨁⨁◯◯ Low	
Anemia	450 (2 cross‐sectional studies)	⨁◯◯◯ Very low	50% of studies were assessed to have an overall poor‐quality rating; 95% CI for OR is wide, sample size 450

Abbreviations: CI, confidence interval; GRADE, Grading of Recommendations Assessment, Development, and Evaluation; OR, odds ratio; SMD, standardized mean difference.

**Table 5:** GRADE of evidence assessment for key review findings for non‐daily versus daily adolescent breakfast consumers.

Outcomes	No. of participants (Studies)	Certainty of the evidence (GRADE)	Reason for downgrading
Overweight/obese	45,111 (10 cross‐sectional studies)	⨁◯◯◯ Very low	40% of studies were assessed to have an overall poor‐quality rating.
Obese	2536 (4 cross‐sectional studies)	⨁⨁◯◯ Low	

Abbreviations: CI, confidence interval; GRADE, Grading of Recommendations Assessment, Development, and Evaluation; OR, odds ratio; SMD, standardized mean difference.

**Table 6:** GRADE of evidence assessment for key review findings for irregular/infrequent versus regular adolescent breakfast consumers.

Outcomes	No. of participants (Studies)	Certainty of the evidence (GRADE)	Reason for downgrading
Overweight	19,230 (3 cross‐sectional studies)	⨁◯◯◯ Very low	*I* ^2^ = 90%, 95% CI spans from [0.95–2.66].
Waist‐height ratio	236 (2 cross‐sectional studies)	⨁⨁◯◯ Low	

Abbreviations: CI, confidence interval; GRADE, Grading of Recommendations Assessment, Development, and Evaluation; OR, odds ratio; SMD, standardized mean difference.

### 6.4 Potential Biases in the Review Process

Review authors made significant efforts to minimize bias throughout the study selection and analysis process. Two independent reviewers conducted the screening and full‐text eligibility phases, data extraction, and quality assessment of the included studies. Discrepancies in study eligibility were resolved through discussion with a third reviewer. Yet, subjectivity may exist during the screening process, introducing the possibility of unintentional biases.

### 6.5 Agreements and Disagreements With Other Studies or Reviews

This is the first meta‐analysis describing the association between breakfast skipping and anthropometric and nutritional outcomes in an adolescent‐specific population, focused on the LMIC context. A review conducted by Ardeshirlarijani et al. of 16 studies (14 cross‐sectional and 2 cohorts) of children and adolescents (5–18 years of age) from both HIC (> 50%) and LMIC contexts found a positive association between breakfast skipping and obesity (1.43, 95% CI: 1.32–1.54 among the cross‐sectional studies (Ardeshirlarijani et al. 2019). In another review of children and adolescents (2–18 years of age) by Monzani et al. 33 studies in HICs and LMICs found a positive association between skipping breakfast and overweight or obesity in 94.7% (270,362/286,804) of subjects included in the review (Monzani et al. 2019). Many studies conducted in youth do not provide separate subgroup analyses for adolescents. Reviews may focus on HIC settings or not explore subgroup LMIC analyses. Furthermore, numerous reviews concentrate on adiposity and anthropometric consequences while failing to address nutrition‐related outcomes despite the nutritional implications of meal skipping, particularly in a population affected by the triple burden of malnutrition.

## 7 Authors' Conclusions

### 7.1 Implications for Practice and Policy

In conclusion, adolescents in low‐resource settings who infrequently and irregularly ate breakfast had 2.05 and 1.32 odds of being overweight/obese, respectively, compared to those who ate breakfast regularly. Those who did not consume breakfast daily had 1.41 odds of being overweight/obese than daily breakfast consumers. Additional measured health outcomes demonstrated a range of both significant and nonsignificant findings but should be interpreted with caution due to high heterogeneity and low sample sizes.

Governments face a significant challenge in addressing the triple burden of malnutrition in adolescents. They must ensure that programs and policies designed to address one form of malnutrition do not exacerbate another. School meal programs are among the most extensive social safety nets for vulnerable children and adolescents globally (World Food Programme [WFP] 2022). Breakfast school meals can be vital in providing students with the nutrients they need to learn and support their health and well‐being. However, school meals alone may not be enough to provide optimal health and nutrition to adolescents. The school environment must be leveraged as a whole to provide a more comprehensive package of interventions, including nutrition education, to encourage adolescents to consume regular and nutritious meals. Breakfast integrated within school feeding programs may be well positioned as a double‐duty solution to tackle malnutrition in all its forms among adolescents (Hawkes et al. 2020).

### 7.2 Implications for Research

It should be restated that definitions of breakfast skipping were highly heterogeneous across studies. We attempted to standardize breakfast frequency into three principal comparisons to compare data across studies. How researchers define and categorize breakfast may significantly influence findings and their interpretation. For example, three studies were excluded because they only collected point prevalence data on whether adolescents had breakfast on the day of the questionnaire, which does not provide insight into habitual breakfast behaviors that may be related to anthropometric or nutritional outcomes (Annan et al. 2020; Gotthelf and Tempestti 2017; Wiafe et al. 2020). This warrants further discussion among the academic community, complemented by future research attempting to establish a standardized definition of what constitutes a breakfast meal and how to categorize breakfast frequency habits in the scientific literature (Hassan et al. 2018). These strides will lessen heterogeneity when pooling findings across studies and will improve interpretations of the existing evidence‐based base informing public health recommendations for adolescents in LMICs.

Although anemia and iron deficiency prevalence were primary outcomes of the present review, only two studies measured them. Iron deficiency anemia is a leading cause of disability‐affected life years (DALY) lost for girls and boys aged 10–14, as well as girls aged 15–19, due to increasing iron needs during this period of rapid growth and development (World Health Organization 2019, 2000–2019). Despite decades of effort to combat this widespread public health challenge, anemia remains a significant problem. Therefore, given this longstanding public health issue and related nutritional challenges, future studies should investigate the relationship between adolescent breakfast habits, iron intake, and absorption, along with other key micronutrients often deficient in this age group. A deeper understanding of these relationships could help develop strategies that not only use breakfast to enhance iron levels but also identify which breakfast foods are most effective for improving iron absorption.

Additional cohort or intervention studies are needed to illuminate the cause‐and‐effect relationship between breakfast skipping and the risk of overweight/obesity among adolescents in low‐ and middle‐income settings. Further, limited studies examine the effects of breakfast skipping on nutritional markers; this area requires additional focus by the nutrition community.

## Author Contributions

Jordie A. J. Fischer and Kesso G. van Zutphen‐Küffer conceived the idea of this project. Jonathan Thomas and Jordie A. J. Fischer drafted the protocol. All other co‐authors provided input, reviewed, and commented on multiple drafts of the protocol, including the following:

- Content: Vanessa Garcia‐Larsen and Kesso G. van Zutphen‐Küffer are experts in the content area;
- Systematic review methods: Vanessa Garcia‐Larsen and Despo Ierodiakonou are experts in systematic review methods;
- Statistical analysis: Despo Ierodiakonou is an expert in meta‐analysis;
- Information retrieval: Vanessa Garcia‐Larsen and Despo Ierodiakonou are experts in information retrieval.

## Conflicts of Interest

The authors declare no conflicts of interest.

## Plans for Updating This Review

The authors do not have plans to update this review at the time of writing.

## Differences Between Protocol and Review

Our review is based on our published protocol (Fischer et al. 2024). We planned to assess the following secondary anthropometry outcomes: height‐for‐age (stunting, *z*‐scores), skinfold thickness, hip circumference, MUAC (10–14 years), and bioelectric impedance. We also planned to assess the following secondary nutritional outcomes: frequency (%) of iron deficiency (defined as serum ferritin < 15 µg/L, as per WHO guidelines) and iron‐deficiency anemia (iron levels as assessed by ferritin or soluble transferrin receptor (sTfR), vitamin A levels as serum/plasma retinol or retinol‐binding protein concentrations, iodine levels as urinary iodine, zinc levels as plasma zinc, and calcium and vitamin D levels as 25(OH)D, parathyroid hormone or total calcium in serum/plasma or urine. However, the abovementioned secondary outcomes were not reported in the 41 included studies. We planned to use the Cochrane risk‐of‐bias tool for randomized trials (RoB 2) to examine potential sources of bias across multiple domains in RCTs and the Risk of Bias in Non‐randomized Studies of Interventions (ROBINS‐*I*) tool to assess the risk of bias in non‐randomized studies. No randomized or non‐randomized intervention studies were included in our review; therefore, these tools were not used. Finally, we planned to investigate possible sources of heterogeneity by performing subgroup analysis on the overall risk of bias and age at outcome, but this data was unavailable. Therefore, we conducted a gender subgroup analysis when outcomes had a sufficient number of studies.

## Sources of Support

### 1 Internal Sources

The authors have no sources of internal support to declare.

### 2 External Sources

The authors have no sources of external support to declare.

## Supporting information

Supporting information.

Supporting information.

## Data Availability

Data will be made available upon reasonable request.
