# Supplementary material for: Breakfast Habits, Anthropometry, and Nutrition‐Related Outcomes in Adolescents From Low‐ and Middle‐Income Countries: A Systematic Review and Meta‐Analysis
Source: Campbell Syst Rev. 2025 Apr 22;21(2):e70039. doi: 10.1002/cl2.70039 (PMC12012571; doi:10.1002/cl2.70039)
Supplement: Supplementary file 1 — Supporting information. [file CL2-21-e70039-s002.pdf]

Summary

Results from search strategy (ran on 6/28):

PubMed	737
EMBASE	1796
CINAHL	110
CENTRAL	118
Web of Science	840
**TOTAL**	**3,601**

Comments:

- Search strategy was based on 3 concepts: breakfast skipping, LMICs, and adolescents
    - Breakfast skipping concept included breakfast terms AND breakfast skipping behavior terms
- LMIC search term was based on a JHU librarian premade filter and other published filters available online

PubMed

Concept #1

Breakfast[mesh] OR breakfast*[tiab] OR "morning meal*"[tiab]

AND

(("Feeding Behavior"[Mesh]) OR (behavio*[Title/Abstract])) OR (skip*[Title/Abstract] OR omit*[Title/Abstract] OR habit*[Title/Abstract] OR pattern*[Title/Abstract])

Concept #2

Adolescent[mesh] OR adolescen*[tiab] OR teen*[tiab] OR youth*[tiab] OR young* OR juvenile OR schoolchild* OR schoolboy* OR schoolgirl* OR student* OR boy OR boys OR girl OR girls OR child*

Concept #3

LMIC filter

"afghanistan"[tiab] OR "albania"[tiab] OR "algeria"[tiab] OR "american samoa"[tiab] OR "angola"[tiab] OR "antigua"[tiab] OR "barbuda"[tiab] OR "argentina"[tiab] OR "armenia"[tiab] OR "armenian"[tiab] OR "aruba"[tiab] OR "azerbaijan"[tiab] OR "bahrain"[tiab] OR "bangladesh"[tiab] OR "barbados"[tiab] OR "belarus"[tiab] OR "byelarus"[tiab] OR "belorussia"[tiab] OR "byelorussian"[tiab] OR "belize"[tiab] OR "british honduras"[tiab] OR "benin"[tiab] OR "dahomey"[tiab] OR "bhutan"[tiab] OR "bolivia"[tiab] OR "bosnia"[tiab] OR "herzegovina"[tiab] OR "botswana"[tiab] OR "bechuanaland"[tiab] OR "brazil"[tiab] OR "brasil"[tiab] OR "bulgaria"[tiab] OR "burkina faso"[tiab] OR "burkina fasso"[tiab] OR "upper volta"[tiab] OR "burundi"[tiab] OR "urundi"[tiab] OR "cabo verde"[tiab] OR "cape verde"[tiab] OR "cambodia"[tiab] OR "kampuchea"[tiab] OR "khmer republic"[tiab] OR "cameroon"[tiab] OR "cameron"[tiab] OR "cameroun"[tiab] OR "central african republic"[tiab] OR "ubangi shari"[tiab] OR "chad"[tiab] OR "chile"[tiab] OR "china"[tiab] OR "colombia"[tiab] OR "comoros"[tiab] OR "comoro islands"[tiab] OR "mayotte"[tiab] OR "congo"[tiab] OR "zaire"[tiab] OR "costa rica"[tiab] OR "cote d ivoire"[tiab] OR "cote d ivoire"[tiab] OR "cote d ivoire"[tiab] OR "ivory coast"[tiab] OR "croatia"[tiab] OR "cuba"[tiab] OR "cyprus"[tiab] OR "czech republic"[tiab] OR "czechoslovakia"[tiab] OR "djibouti"[tiab] OR "french somaliland"[tiab] OR "dominica"[tiab] OR "dominican republic"[tiab] OR "ecuador"[tiab] OR "egypt"[tiab] OR "united arab republic"[tiab] OR "el salvador"[tiab] OR "equatorial guinea"[tiab] OR "spanish guinea"[tiab] OR "eritrea"[tiab] OR "estonia"[tiab] OR "eswatini"[tiab] OR "swaziland"[tiab] OR "ethiopia"[tiab] OR "fiji"[tiab] OR "gabon"[tiab] OR "gabonese republic"[tiab] OR "gambia"[tiab] OR "georgia"[tiab] OR "georgian"[tiab] OR "ghana"[tiab] OR "gold coast"[tiab] OR "gibraltar"[tiab] OR "grenada"[tiab] OR "guam"[tiab]

OR "guatemala"[tiab] OR "guinea"[tiab] OR "guyana"[tiab] OR "guiana"[tiab] OR "haiti"[tiab] OR "hispaniola"[tiab] OR "honduras"[tiab] OR "india"[tiab] OR "indonesia"[tiab] OR "timor"[tiab] OR "iran"[tiab] OR "iraq"[tiab] OR "isle of man"[tiab] OR "jamaica"[tiab] OR "jordan"[tiab] OR "kazakhstan"[tiab] OR "kazakh"[tiab] OR "kenya"[tiab] OR "korea"[tiab] OR "kosovo"[tiab] OR "kyrgyzstan"[tiab] OR "kirghizia"[tiab] OR "kirgizstan"[tiab] OR "kyrgyz republic"[tiab] OR "kirghiz"[tiab] OR "laos"[tiab] OR "lao pdr"[tiab] OR "lao people s democratic republic"[tiab] OR "latvia"[tiab] OR "lebanon"[tiab] OR "lesotho"[tiab] OR "basutoland"[tiab] OR "liberia"[tiab] OR "libya"[tiab] OR "libyan arab jamahiriya"[tiab] OR "lithuania"[tiab] OR "macau"[tiab] OR "macao"[tiab] OR "macedonia"[tiab] OR "madagascar"[tiab] OR "malagasy republic"[tiab] OR "malawi"[tiab] OR "nyasaland"[tiab] OR "malaysia"[tiab] OR "maldives"[tiab] OR "indian ocean"[tiab] OR "mali"[tiab] OR "malta"[tiab] OR "micronesia"[tiab] OR "kiribati"[tiab] OR "marshall islands"[tiab] OR "nauru"[tiab] OR "northern mariana islands"[tiab] OR "palau"[tiab] OR "tuvalu"[tiab] OR "mauritania"[tiab] OR "mauritius"[tiab] OR "mexico"[tiab] OR "moldova"[tiab] OR "moldovian"[tiab] OR "mongolia"[tiab] OR "montenegro"[tiab] OR "morocco"[tiab] OR "ifni"[tiab] OR "mozambique"[tiab] OR "portuguese east africa"[tiab] OR "myanmar"[tiab] OR "burma"[tiab] OR "namibia"[tiab] OR "nepal"[tiab] OR "netherlands antilles"[tiab] OR "nicaragua"[tiab] OR "niger"[tiab] OR "nigeria"[tiab] OR "oman"[tiab] OR "muscat"[tiab] OR "pakistan"[tiab] OR "panama"[tiab] OR "papua new guinea"[tiab] OR "paraguay"[tiab] OR "peru"[tiab] OR "philippines"[tiab] OR "philipines"[tiab] OR "phillipines"[tiab] OR "phillippines"[tiab] OR "poland"[tiab] OR "polish people s republic"[tiab] OR "portugal"[tiab] OR "portuguese republic"[tiab] OR "puerto rico"[tiab] OR "romania"[tiab] OR "russia"[tiab] OR "russian federation"[tiab] OR "ussr"[tiab] OR "soviet union"[tiab] OR "union of soviet socialist republics"[tiab] OR "rwanda"[tiab] OR "ruanda"[tiab] OR "samoa"[tiab] OR "pacific islands"[tiab] OR "polynesia"[tiab] OR "samoan islands"[tiab] OR "sao tome and principe"[tiab] OR "saudi arabia"[tiab] OR "senegal"[tiab] OR "serbia"[tiab] OR "seychelles"[tiab] OR "sierra leone"[tiab] OR "slovakia"[tiab] OR "slovak republic"[tiab] OR "slovenia"[tiab] OR "melanesia"[tiab] OR "solomon island"[tiab] OR "solomon islands"[tiab] OR "norfolk island"[tiab] OR "somalia"[tiab] OR "south africa"[tiab] OR "south sudan"[tiab] OR "sri lanka"[tiab] OR "ceylon"[tiab] OR "saint kitts and nevis"[tiab] OR "st kitts and nevis"[tiab] OR "saint lucia"[tiab] OR "st lucia"[tiab] OR "saint vincent"[tiab] OR "st vincent"[tiab] OR "grenadines"[tiab] OR "sudan"[tiab] OR "suriname"[tiab] OR "surinam"[tiab] OR "syria"[tiab] OR "syrian arab republic"[tiab] OR "tajikistan"[tiab] OR "tadjikistan"[tiab] OR "tadzhikistan"[tiab] OR "tadzhik"[tiab] OR "tanzania"[tiab] OR "tanganyika"[tiab] OR "thailand"[tiab] OR "siam"[tiab] OR "timor leste"[tiab] OR "east timor"[tiab] OR "togo"[tiab] OR "togolese republic"[tiab] OR "tonga"[tiab] OR "trinidad"[tiab] OR "tobago"[tiab] OR "tunisia"[tiab] OR "turkey"[tiab] OR "turkmenistan"[tiab] OR "turkmen"[tiab] OR "uganda"[tiab] OR "ukraine"[tiab] OR "uruguay"[tiab] OR "uzbekistan"[tiab] OR "uzbek"[tiab] OR "vanuatu"[tiab] OR "new hebrides"[tiab] OR "venezuela"[tiab] OR "vietnam"[tiab] OR "viet nam"[tiab] OR "middle east"[tiab] OR "west bank"[tiab] OR "gaza"[tiab] OR "palestine"[tiab] OR "yemen"[tiab] OR "yugoslavia"[tiab] OR "zambia"[tiab] OR "zimbabwe"[tiab] OR "northern rhodesia"[tiab] OR "global south"[tiab] OR "africa south of the sahara"[tiab] OR "sub saharan africa"[tiab] OR "subsaharan africa"[tiab] OR "central africa"[tiab] OR "north africa"[tiab] OR "northern africa"[tiab] OR "magreb"[tiab] OR "maghrib"[tiab] OR "sahara"[tiab] OR "southern africa"[tiab] OR "east africa"[tiab] OR "eastern africa"[tiab] OR "west africa"[tiab] OR "western africa"[tiab] OR "west indies"[tiab] OR "indian ocean islands"[tiab] OR "caribbean"[tiab] OR

"central america"[tiab] OR "latin america"[tiab] OR "south america"[tiab] OR "central asia"[tiab] OR "north asia"[tiab] OR "northern asia"[tiab] OR "southeastern asia"[tiab] OR "south eastern asia"[tiab] OR "southeast asia"[tiab] OR "south east asia"[tiab] OR "western asia"[tiab] OR "east europe"[tiab] OR "eastern europe"[tiab] OR "developing country"[tiab] OR "developing countries"[tiab] OR "developing nation"[tiab] OR "developing nations"[tiab] OR "developing population"[tiab] OR "developing populations"[tiab] OR "developing world"[tiab] OR "less developed country"[tiab] OR "less developed countries"[tiab] OR "less developed nation"[tiab] OR "less developed nations"[tiab] OR "less developed world"[tiab] OR "lesser developed countries"[tiab] OR "lesser developed nations"[tiab] OR "under developed country"[tiab] OR "under developed countries"[tiab] OR "under developed nations"[tiab] OR "under developed world"[tiab] OR "underdeveloped country"[tiab] OR "underdeveloped countries"[tiab] OR "underdeveloped nation"[tiab] OR "underdeveloped nations"[tiab] OR "underdeveloped population"[tiab] OR "underdeveloped populations"[tiab] OR "underdeveloped world"[tiab] OR "middle income country"[tiab] OR "middle income countries"[tiab] OR "middle income nation"[tiab] OR "middle income nations"[tiab] OR "middle income population"[tiab] OR "middle income populations"[tiab] OR "low income country"[tiab] OR "low income countries"[tiab] OR "low income nation"[tiab] OR "low income nations"[tiab] OR "low income population"[tiab] OR "low income populations"[tiab] OR "lower income country"[tiab] OR "lower income countries"[tiab] OR "lower income nations"[tiab] OR "lower income population"[tiab] OR "lower income populations"[tiab] OR "underserved countries"[tiab] OR "underserved nations"[tiab] OR "underserved population"[tiab] OR "underserved populations"[tiab] OR "under served population"[tiab] OR "under served populations"[tiab] OR "deprived countries"[tiab] OR "deprived population"[tiab] OR "deprived populations"[tiab] OR "poor country"[tiab] OR "poor countries"[tiab] OR "poor nation"[tiab] OR "poor nations"[tiab] OR "poor population"[tiab] OR "poor populations"[tiab] OR "poor world"[tiab] OR "poorer countries"[tiab] OR "poorer nations"[tiab] OR "poorer population"[tiab] OR "poorer populations"[tiab] OR "developing economy"[tiab] OR "developing economies"[tiab] OR "less developed economy"[tiab] OR "less developed economies"[tiab] OR "underdeveloped economies"[tiab] OR "middle income economy"[tiab] OR "middle income economies"[tiab] OR "low income economy"[tiab] OR "low income economies"[tiab] OR "lower income economies"[tiab] OR "low gdp"[tiab] OR "low gnp"[tiab] OR "low gross domestic"[tiab] OR "low gross national"[tiab] OR "lower gdp"[tiab] OR "lower gross domestic"[tiab] OR "lmic"[tiab] OR "lmics"[tiab] OR "third world"[tiab] OR "lami country"[tiab] OR "lami countries"[tiab] OR "transitional country"[tiab] OR "transitional countries"[tiab] OR "emerging economies"[tiab] OR "emerging nation"[tiab] OR "emerging nations"[tiab] OR "afghanistan"[mesh] OR "albania"[mesh] OR "algeria"[mesh] OR "american samoa"[mesh] OR "angola"[mesh] OR "antigua and barbuda"[mesh] OR "argentina"[mesh] OR "armenia"[mesh] OR "aruba"[mesh] OR "azerbaijan"[mesh] OR "bahrain"[mesh] OR "bangladesh"[mesh] OR "barbados"[mesh] OR "republic of belarus"[mesh] OR "belize"[mesh] OR "benin"[mesh] OR "bhutan"[mesh] OR "bolivia"[mesh] OR "bosnia and herzegovina"[mesh] OR "botswana"[mesh] OR "brazil"[mesh] OR "bulgaria"[mesh] OR "burkina faso"[mesh] OR "burundi"[mesh] OR "cabo verde"[mesh] OR "cambodia"[mesh] OR "cameroon"[mesh] OR "central african republic"[mesh] OR "chad"[mesh] OR "chile"[mesh] OR "china"[mesh] OR "colombia"[mesh] OR "comoros"[mesh] OR "democratic republic of the congo"[mesh] OR "congo"[mesh] OR "costa rica"[mesh] OR "cote d ivoire"[mesh] OR "croatia"[mesh] OR "cuba"[mesh] OR "cyprus"[mesh] OR "czech republic"[mesh] OR "djibouti"[mesh] OR "dominica"[mesh] OR

"dominican republic"[mesh] OR "ecuador"[mesh] OR "egypt"[mesh] OR "el salvador"[mesh] OR "equatorial guinea"[mesh] OR "eritrea"[mesh] OR "estonia"[mesh] OR "eswatini"[mesh] OR "ethiopia"[mesh] OR "fiji"[mesh] OR "gabon"[mesh] OR "gambia"[mesh] OR "georgia republic"[mesh] OR "ghana"[mesh] OR "gibraltar"[mesh] OR "grenada"[mesh] OR "guam"[mesh] OR "guatemala"[mesh] OR "guinea"[mesh] OR "guinea bissau"[mesh] OR "guyana"[mesh] OR "haiti"[mesh] OR "honduras"[mesh] OR "india"[mesh] OR "indonesia"[mesh] OR "iran"[mesh] OR "iraq"[mesh] OR "jamaica"[mesh] OR "jordan"[mesh] OR "kazakhstan"[mesh] OR "kenya"[mesh] OR "democratic people s republic of korea"[mesh] OR "republic of korea"[mesh] OR "kosovo"[mesh] OR "kyrgyzstan"[mesh] OR "laos"[mesh] OR "latvia"[mesh] OR "lebanon"[mesh] OR "lesotho"[mesh] OR "liberia"[mesh] OR "libya"[mesh] OR "lithuania"[mesh] OR "macau"[mesh] OR "republic of north macedonia"[mesh] OR "madagascar"[mesh] OR "malawi"[mesh] OR "malaysia"[mesh] OR "indian ocean islands"[mesh] OR "mali"[mesh] OR "malta"[mesh] OR "micronesia"[mesh] OR "palau"[mesh] OR "mauritania"[mesh] OR "mauritius"[mesh] OR "mexico"[mesh] OR "moldova"[mesh] OR "mongolia"[mesh] OR "montenegro"[mesh] OR "morocco"[mesh] OR "mozambique"[mesh] OR "myanmar"[mesh] OR "namibia"[mesh] OR "nepal"[mesh] OR "netherlands antilles"[mesh] OR "nicaragua"[mesh] OR "niger"[mesh] OR "nigeria"[mesh] OR "oman"[mesh] OR "pakistan"[mesh] OR "panama"[mesh] OR "papua new guinea"[mesh] OR "paraguay"[mesh] OR "peru"[mesh] OR "philippines"[mesh] OR "poland"[mesh] OR "portugal"[mesh] OR "puerto rico"[mesh] OR "romania"[mesh] OR "russia"[mesh] OR "rwanda"[mesh] OR "samoa"[mesh] OR "sao tome and principe"[mesh] OR "saudi arabia"[mesh] OR "senegal"[mesh] OR "serbia"[mesh] OR "seychelles"[mesh] OR "sierra leone"[mesh] OR "slovakia"[mesh] OR "slovenia"[mesh] OR "melanesia"[mesh] OR "somalia"[mesh] OR "south africa"[mesh] OR "south sudan"[mesh] OR "sri lanka"[mesh] OR "saint kitts and nevis"[mesh] OR "saint lucia"[mesh] OR "saint vincent and the grenadines"[mesh] OR "sudan"[mesh] OR "suriname"[mesh] OR "syria"[mesh] OR "tajikistan"[mesh] OR "tanzania"[mesh] OR "thailand"[mesh] OR "timor leste"[mesh] OR "togo"[mesh] OR "tonga"[mesh] OR "trinidad and tobago"[mesh] OR "tunisia"[mesh] OR "turkey"[mesh] OR "turkmenistan"[mesh] OR "uganda"[mesh] OR "ukraine"[mesh] OR "uruguay"[mesh] OR "uzbekistan"[mesh] OR "vanuatu"[mesh] OR "venezuela"[mesh] OR "vietnam"[mesh] OR "middle east"[mesh] OR "yemen"[mesh] OR "yugoslavia"[mesh] OR "zambia"[mesh] OR "zimbabwe"[mesh] OR "africa south of the sahara"[mesh] OR "africa, central"[mesh] OR "africa, northern"[mesh] OR "africa, southern"[mesh] OR "africa, eastern"[mesh] OR "africa, western"[mesh] OR "west indies"[mesh] OR "indian ocean islands"[mesh] OR "caribbean region"[mesh] OR "central america"[mesh] OR "latin america"[mesh] OR "south america"[mesh] OR "asia, central"[mesh] OR "asia, northern"[mesh] OR "asia, southeastern"[mesh] OR "asia, western"[mesh] OR "europe, eastern"[mesh] OR "developing countries"[mesh]

RESULTS: #1 AND #2 AND #3 – **944 records**

- with Human and English filter = **737 records**

EMBASE

Embase <1980 to 2023 June 28>

1 exp meal/ 26092

2 breakfast*.mp. 17137

3 morning meal.mp. 268

4 1 or 2 or 3 35029

5 (afghan* or africa* or albania* or algeria* or angola* or antigua* or barbuda* or argentin* or armenia* or aruba* or azerbaijan* or bahrain* or bangladesh* or bengal* or bangal* or barbados* or barbadian* or bajan or bajans or belarus* or belorus* or byelarus* or byelorus* or belize* or benin* or dahomey or bhutan* or bolivia* or bosnia* or herzegovin* or botswan* or batswan* or bechuanaland* or brazil* or brasil* or bulgaria* or burkina* or burkinese* or upper volta* or burundi* or urundi* or cabo verde* or cape verde* or cambodia* or kampuchea* or khmer* or cameroon* or cameroun* or ubangi shari* or chad* or chile* or china* or chinese or colombia* or comoro* or comore* or comorian* or mayotte* or congo* or zaire* or costa rica* or "cote d'ivoir*" or "cote d' ivoir*" or cote divoir* or cote d ivoir* or ivory coast* or ivorian* or croatia* or cuba or cuban or cubans or "cuba's" or cyprus* or cypriot* or czech* or djibouti* or french somaliland* or dominica* or ecuador* or egypt* or united arab republic* or el salvador* or salvadoran* or guinea* or equatoguinea* or eritrea* or estonia* or eswatini* or swaziland* or swazi* or swati* or ethiopia* or fiji* or gabon* or gabonese* or gabonaise* or gambia* or ((georgia or georgian or georgians) not (atlanta or california or florida)) or ghana* or gibraltar* or greece* or greek* or grecian* or grenada* or grenadian* or guam* or guatemala* or guyana* or guiana* or guyanese* or haiti* or hispaniola* or hondura* or hungary* or hungarian* or india* or indonesia* or iran* or iraq* or isle of man* or jamaica* or jordan* or kazakh* or kenya* or karabati* or korea* or kosovo* or kosova* or kyrgyz* or kirgiz* or kirghiz* or laos or lao or laotian* or latvia* or lebanon* or lebanese* or lesotho* or lesothan* or lesothonian* or basutoland* or mosotho* or basotho* or liberia* or libya* or jamahiriya* or lithuania* or macedonia* or madagasca* or malagasy* or malawi* or nyasaland* or malaysia* or malay* federation or maldives* or maldivian* or indian ocean or mali or malian* or "mali's" or malta or maltese* or "malta's" or micronesia* or marshallese* or kiribati* or marshall island* or nauru or nauran or nauruans or "naurian's" or mariana or marianas or palau or paluan* or tuvalu* or mauritania* or mauritan* or mauritius* or mexico* or mexican* or moldova* or moldovia* or mongol* or montenegr* or morocco* or moroccan* or ifni or mozambique* or mozambican* or myanmar* or burma* or burmese or namibia* or nepal* or new caledonia* or netherlands antill* or nicaragua* or niger* or oman or omani or omanis or "oman's" or pakistan* or palestin* or gaza* or west bank* or panama* or paraguay* or peru or peruvian* or "peru's" or philippine* or philipine* or phillipine* or phillippine* or filipino* or filipina* or poland* or polish or pole or poles or portugal* or portuguese or puerto ric* or romania* or russia* or ussr* or soviet* or rwanda* or rwandese or ruanda* or ruandese or samoa* or navigator island* or pacific island* or polynesia* or "sao tome and principe*" or sao tomean* or santomean* or saudi arabia* or saudi or saudis or senegal* or serbia* or seychell* or sierra leone* or slovak* or sloven* or melanesia* or solomon island* or norfolk island* or somali* or sri lanka* or ceylon* or "saint kitts and nevis*" or "st

kitts and nevis*" or kittian* or nevisian* or saint lucia* or st lucia* or saint vincent* or st vincent* or vincentian* or grenadine* or sudan* or surinam* or syria* or tajik* or tadjik* or tadzhik* or tanzania* or tanganyika* or thai* or timor leste* or east timor* or timorese* or togo or togoles* or "togo's" or tonga* or trinidad* or tobago* or tunisia* or turkiy* or turkey* or turk or turks or turkish or turkmen* or uganda* or ukrain* or uruguay* or uzbek* or vanuatu* or new hebrides* or venezuela* or vietnam* or viet nam* or yemen* or yugoslav* or zambia* or zimbabwe* or rhodesia* or arab* countr* or middle east* or global south or sahara* or subsahara* or magreb* or maghrib* or west indies* or caribbean* or central america* or latin america* or south america* or central asia* or north asia* or northern asia* or southeastern asia* or south eastern asia* or southeast asia* or south east asia* or west asia* or western asia* or east europe* or eastern europe* or developing countr* or developing nation* or developing population* or developing world or less developed countr* or less developed nation* or less developed world or lesser developed countr* or lesser developed nation* or lesser developed world or under developed countr* or under developed nation* or under developed world or underdeveloped countr* or underdeveloped nation* or underdeveloped world or middle income countr* or middle income nation* or middle income population* or low income countr* or low income nation* or low income population* or lower income countr* or lower income nation* or lower income population* or underserved countr* or underserved nation* or underserved population* or under served population* or under served nation* or under served population* or deprived countr* or deprived population* or high burden countr* or high burden nation* or countdown countr* or countdown nation* or poor countr* or poor nation* or poor population* or poor world or poorer countr* or poorer nation* or poorer population* or poorer world or developing econom* or less developed econom* or underdeveloped econom* or under developed econom* or middle income econom* or low income econom* or lower income econom* or low gdp or low gnp or low gross domestic or low gross national or lower gdp or lower gnp or lower gross domestic or lower gross national or lmic or lmics or third world or lami countr* or transitional countr* or emerging econom* or emerging nation*).ti,ab,hw,kf,jx. 4704349

6 exp adolescent/ 1717618

7 exp child/ 2944138

8 exp juvenile/ or exp child/ 3864150

9 young adult/ 510713

10 exp school child/ 412528

11 student/ or exp elementary student/ or exp high school student/ or exp middle school student/ or exp university student/ 146605

12 exp boy/ 28429

13 exp girl/ 40111

14 (adolescen* or child*).mp. 3849151

15 teen*.mp. 49137

16 youth*.mp. 124071

17 young*.mp. 1503280

18 juvenile.mp. 164192

19 schoolchild*.mp. 17732

20 schoolboy*.mp. 507

21 schoolgirl*.mp. 915

22 student*.mp. 599228

23 (boy or boys).mp. 220193

24 girl*.mp. 226940

25 6 or 7 or 8 or 9 or 10 or 11 or 12 or 13 or 14 or 15 or 16 or 17 or 18 or 19 or 20 or 21 or 22 or 23 or 24 5873228

26 4 and 5 and 25 2991

27 skip*.mp. 22942

28 omit*.mp. 33410

29 habit*.mp. or habit/ 293840

30 feeding behavior/ or eating habit/ 99666

31 behavio*.mp. 2346564

32 pattern*.mp. or exp dietary pattern/ 1872883

33 27 or 28 or 29 or 30 or 31 or 32 4251121

34 4 and 33 11609

35 5 and 25 and 341944

36 limit 35 to english language 1800

37 (animal or animals or canine* or dog or dogs or feline or hamster* or lamb or lambs or mice or monkey or monkeys or mouse or murine or pig or pigs or piglet* or porcine or primate* or rabbit* or rats or rat or rodent* or sheep* or veterinar*).ti,kw,dq,jx. not (human* or patient*).mp. 1993278

38 (exp animal/ or exp juvenile animal/ or adult animal/ or animal cell/ or animal tissue/ or nonhuman/ or animal experiment/ or animal model/) not human/ 6908526

39 36 not (37 or 38) 1796

40 limit 39 to english language **1796**

CINAHL

Thursday, June 29, 2023 3:36:55 AM

#	Query	Limiters/Expanders	Last Run Via	Results
S31	S7 AND S21 AND S30	Expanders - Apply equivalent subjects Search modes - Boolean/Phrase	Interface - EBSCOhost Research Databases Search Screen - Advanced Search Database - CINAHL Ultimate	110
S30	S4 AND S29	Expanders - Apply equivalent subjects Search modes - Boolean/Phrase	Interface - EBSCOhost Research Databases Search Screen - Advanced Search Database - CINAHL Ultimate	2,713
S29	S23 OR S24 OR S25 OR S26 OR S27 OR S28	Expanders - Apply equivalent subjects Search modes - Boolean/Phrase	Interface - EBSCOhost Research Databases Search Screen - Advanced Search Database - CINAHL Ultimate	790,878
S28	pattern*	Expanders - Apply equivalent subjects Search modes - Boolean/Phrase	Interface - EBSCOhost Research Databases Search Screen - Advanced Search Database - CINAHL Ultimate	238,325
S27	habit*	Expanders - Apply equivalent subjects Search modes - Boolean/Phrase	Interface - EBSCOhost Research Databases Search Screen - Advanced Search Database - CINAHL Ultimate	83,075
S26	omit*	Expanders - Apply equivalent subjects Search modes - Boolean/Phrase	Interface - EBSCOhost Research Databases Search Screen - Advanced Search Database - CINAHL Ultimate	8,941
S25	skip*	Expanders - Apply equivalent subjects Search modes - Boolean/Phrase	Interface - EBSCOhost Research Databases Search Screen - Advanced	4,124

			Search<br>Database - CINAHL Ultimate	
S24	behavio*	Expanders - Apply equivalent subjects<br>Search modes - Boolean/Phrase	Interface - EBSCOhost Research Databases<br>Search Screen - Advanced Search<br>Database - CINAHL Ultimate	523,414
S23	(MH "Food Habits") OR (MH "Eating Behavior")	Expanders - Apply equivalent subjects<br>Search modes - Boolean/Phrase	Interface - EBSCOhost Research Databases<br>Search Screen - Advanced Search<br>Database - CINAHL Ultimate	36,144
S22	S8 AND S21	Expanders - Apply equivalent subjects<br>Search modes - Boolean/Phrase	Interface - EBSCOhost Research Databases<br>Search Screen - Advanced Search<br>Database - CINAHL Ultimate	136
S21	S9 OR S10 OR S11 OR S12 OR S13 OR S14 OR S15 OR S16 OR S17 OR S18 OR S19 OR S20	Expanders - Apply equivalent subjects<br>Search modes - Boolean/Phrase	Interface - EBSCOhost Research Databases<br>Search Screen - Advanced Search<br>Database - CINAHL Ultimate	1,871,808
S20	"girl*"	Expanders - Apply equivalent subjects<br>Search modes - Boolean/Phrase	Interface - EBSCOhost Research Databases<br>Search Screen - Advanced Search<br>Database - CINAHL Ultimate	75,288
S19	"boy" OR "boys"	Expanders - Apply equivalent subjects<br>Search modes - Boolean/Phrase	Interface - EBSCOhost Research Databases<br>Search Screen - Advanced Search<br>Database - CINAHL Ultimate	46,477
S18	"student*" OR (MH "Students") OR (MH "Students, Elementary") OR (MH "Students, Middle School") OR (MH "Students, High School")	Expanders - Apply equivalent subjects<br>Search modes - Boolean/Phrase	Interface - EBSCOhost Research Databases<br>Search Screen - Advanced Search<br>Database - CINAHL Ultimate	286,145

S17	"schoolgirl*"	Expanders - Apply equivalent subjects<br>Search modes - Boolean/Phrase	Interface - EBSCOhost Research Databases<br>Search Screen - Advanced Search<br>Database - CINAHL Ultimate	282
S16	"schoolboy*"	Expanders - Apply equivalent subjects<br>Search modes - Boolean/Phrase	Interface - EBSCOhost Research Databases<br>Search Screen - Advanced Search<br>Database - CINAHL Ultimate	121
S15	"schoolchild"	Expanders - Apply equivalent subjects<br>Search modes - Boolean/Phrase	Interface - EBSCOhost Research Databases<br>Search Screen - Advanced Search<br>Database - CINAHL Ultimate	3,627
S14	"juvenile"	Expanders - Apply equivalent subjects<br>Search modes - Boolean/Phrase	Interface - EBSCOhost Research Databases<br>Search Screen - Advanced Search<br>Database - CINAHL Ultimate	17,386
S13	(MH "Young Adult") OR "young*"	Expanders - Apply equivalent subjects<br>Search modes - Boolean/Phrase	Interface - EBSCOhost Research Databases<br>Search Screen - Advanced Search<br>Database - CINAHL Ultimate	548,070
S12	"youth*"	Expanders - Apply equivalent subjects<br>Search modes - Boolean/Phrase	Interface - EBSCOhost Research Databases<br>Search Screen - Advanced Search<br>Database - CINAHL Ultimate	68,981
S11	"teen*"	Expanders - Apply equivalent subjects<br>Search modes - Boolean/Phrase	Interface - EBSCOhost Research Databases<br>Search Screen - Advanced Search<br>Database - CINAHL Ultimate	26,826
S10	"adolescen*" OR "child*"	Expanders - Apply equivalent subjects<br>Search modes - Boolean/Phrase	Interface - EBSCOhost Research Databases<br>Search Screen - Advanced	1,296,588

			Search<br>Database - CINAHL Ultimate	
S9	(MH "Adolescence+") OR (MH "Child+") OR (MH "Minors (Legal)")	Expanders - Apply equivalent subjects<br>Search modes - Boolean/Phrase	Interface - EBSCOhost Research Databases<br>Search Screen - Advanced Search<br>Database - CINAHL Ultimate	1,117,706
S8	S4 AND S7	Expanders - Apply equivalent subjects<br>Search modes - Boolean/Phrase	Interface - EBSCOhost Research Databases<br>Search Screen - Advanced Search<br>Database - CINAHL Ultimate	183
S7	S5 OR S6	Expanders - Apply equivalent subjects<br>Search modes - Boolean/Phrase	Interface - EBSCOhost Research Databases<br>Search Screen - Advanced Search<br>Database - CINAHL Ultimate	190,466
S6	TI ("cabo verde*" or "democratic people's republic of korea" or eswatini or kiribati* or "gaza strip" or grenada or grenadian* or grenadines or kosov* or "lao people's democratic republic" or maldiv* or "marshall island*" or mauriti* or montenegr* or montserrat* or nauru* or niue* or palestin* or "saint helena*" or "saint lucia*" or "saint vincent*" or "sao tome*" or "solomon island*" or "south sudan*" or timorese or tokelau* or tonga* or tuvalu* or vanuatu* or "wallis and futuna" or "west bank") OR AB ("cabo verde*" or "democratic people's republic of korea" or	Expanders - Apply equivalent subjects<br>Search modes - Boolean/Phrase	Interface - EBSCOhost Research Databases<br>Search Screen - Advanced Search<br>Database - CINAHL Ultimate	4,996

	eswatini or kiribati* or "gaza strip" or grenada or grenadian* or grenadines or kosov* or "lao people's democratic republic" or maldiv* or "marshall island*" or mauriti* or montenegr* or montserrat* or nauru* or niue* or palestin* or "saint helena*" or "saint lucia*" or "saint vincent*" or "sao tome*" or "solomon island*" or "south sudan*" or timorese or tokelau* or tonga* or tuvalu* or vanuatu* or "wallis and futuna" or "west bank")			
S5	MH "Developing Countries" or MH "low and middle income countries" or MH "Afghanistan" or MH "Albania" or MH "Algeria" or MH "Angola" or MH "Argentina" or MH "Armenia" or MH "Azerbaijan" or MH "Bangladesh" or MH "Belarus" or MH "Belize" or MH "Benin" or MH "Bhutan" or MH "Bolivia" or MH "Bosnia-Herzegovina" or MH "Botswana" or MH "Brazil" or MH "Burkina Faso" or MH "Burundi" or MH "Cambodia" or MH "Cameroon" or MH "Cape Verde" or MH "Central African Republic" or MH "Chad" or MH "China" or MH "Colombia" or MH "Comoros" or MH "Congo"	Expanders - Apply equivalent subjects<br>Search modes - Boolean/Phrase	Interface - EBSCOhost Research Databases<br>Search Screen - Advanced Search<br>Database - CINAHL Ultimate	185,803

or MH "Costa Rica" or MH "Cote d'Ivoire" or MH "Cuba" or MH "Democratic Republic of Congo" or MH "Djibouti" or MH "Dominica" or MH "Dominican Republic" or MH "East Timor" or MH "Ecuador" or MH "Egypt" or MH "El Salvador" or MH "Equatorial Guinea" or MH "Eritrea" or MH "Ethiopia" or MH "Fiji" or MH "Gabon" or MH "Gambia" or MH "Georgia (Republic)" or MH "Ghana" or MH "Guatemala" or "MH "Guinea" or MH "Guinea-Bissau" or MH "Guyana" or MH "Haiti" or MH "Honduras" or MH "India" or MH "Indian Ocean Islands" or MH "Indonesia" or MH "Iran" or MH "Iraq" or MH "Jamaica" or MH "Jordan" or MH "Kazakhstan" or MH "Kenya" or MH "Kyrgyzstan" or MH "Laos" or MH "Lebanon" or MH "Lesotho" or MH "Liberia" or MH "Libya" or MH "Macedonia (Republic)" or MH "Madagascar" or MH "Malawi" or MH "Malaysia" or MH "Mali" or MH "Mauritania" or MH "Mexico" or MH "Micronesia" or MH "Moldova" or MH "Mongolia" or MH "Morocco" or MH

	"Mozambique" or MH "Myanmar" or MH "Namibia" or MH "Nepal" or MH "Nicaragua" or MH "Niger" or MH "Nigeria" or MH "North Korea" or MH "Pakistan" or MH "Panama" or MH "Papua New Guinea" or MH "Paraguay" or MH "Peru" or MH "Philippines" or MH "Polynesia" or MH "Rwanda" or MH "Samoa" or MH "Senegal" or MH "Serbia" or MH "Sierra Leone" or MH "Somalia" or MH "South Africa" or MH "Sri Lanka" or MH "Sudan" or MH Suriname or MH "Swaziland" or MH "Syria" or MH "Tajikistan" or MH "Tanzania" or MH "Thailand" or MH "Togo" or MH "Tunisia" or MH "Turkey" or MH "Turkmenistan" or MH "Uganda" or MH "Ukraine" or MH "Uzbekistan" or MH "Venezuela" or MH "Vietnam" or MH "Yemen" or MH "Zambia" or MH "Zimbabwe"			
S4	S1 OR S2 OR S3	Expanders - Apply equivalent subjects<br>Search modes - Boolean/Phrase	Interface - EBSCOhost Research Databases<br>Search Screen - Advanced Search<br>Database - CINAHL Ultimate	6,332
S3	"morning meal"	Expanders - Apply equivalent subjects<br>Search modes - Boolean/Phrase	Interface - EBSCOhost Research Databases<br>Search Screen - Advanced Search<br>Database - CINAHL Ultimate	1,456

S2	"breakfast*"	Expanders - Apply equivalent subjects<br>Search modes - Boolean/Phrase	Interface - EBSCOhost Research Databases<br>Search Screen - Advanced Search<br>Database - CINAHL Ultimate	6,305
S1	(MH "Breakfast")	Expanders - Apply equivalent subjects<br>Search modes - Boolean/Phrase	Interface - EBSCOhost Research Databases<br>Search Screen - Advanced Search<br>Database - CINAHL Ultimate	2,169

CENTRAL

- Search Name:
- Date Run: 28/06/2023 05:52:50
- Comment:
-
- ID Search Hits
- #1 MeSH descriptor: [Breakfast] explode all trees 423
- #2 (breakfast*):ti,ab,kw 8372
- #3 (morning meal*):ti,ab,kw 2096
- #4 (afghan* OR africa* OR albania* OR algeria* OR angola* OR antigua* OR barbuda* OR argentin* OR armenia* OR aruba* OR azerbaijan* OR bahrain* OR bangladesh* OR bengal* OR bangal* OR barbados* OR barbadian* OR bajan OR bajans OR belarus* OR belorus* OR byelarus* OR byelorus* OR belize* OR benin* OR dahomey OR bhutan* OR bolivia* OR bosnia* OR herzegovin* OR botswan* OR batswan* OR bechuanaland OR brazil* OR brasil* OR bulgaria* OR burkina* OR burkinese* OR upper-volta* OR burundi* OR urundi* OR cabo-verde* OR cape-verde* OR cambodia* OR kampuchea* OR khmer* OR cameroon* OR cameroun* OR ubangi-shari* OR chad* OR chile* OR china* OR chinese OR colombia* OR comoro* OR comore* OR comorian* OR mayotte* OR congo* OR zaire* OR costa-rica* OR (cote* AND *ivoir*) OR ivory-coast* OR ivorian* OR croatia* OR cuba* OR cyprus* OR cypriot* OR czech* OR djibouti* OR french-somaliland* OR dominica* OR ecuador* OR egypt* OR united-arab-republic* OR el-salvador* OR salvadoran* OR guinea* OR equatoguinea* OR eritrea* OR estonia* OR eswatini* OR swaziland* OR swazi* OR swati* OR ethiopia* OR fiji* OR gabon* OR gabonese* OR gabonaise* OR gambia* OR ((georgia OR georgian OR georgians) NOT (atlanta OR california OR florida)) OR ghana* OR gibraltar* OR greece* OR greek* OR grecian* OR grenada* OR grenadian* OR guam* OR guatemala* OR guyana* OR guiana* OR guyanese* OR haiti* OR hispaniola* OR hondura* OR hungary* OR hungarian* OR india* OR indonesia* OR iran* OR iraq* OR isle-of-man* OR jamaica* OR jordan* OR kazakh* OR kenya* OR karabati* OR korea* OR kosovo* OR kosova* OR kyrgyz* OR kirgiz* OR kirghiz* OR laos OR lao OR laotian* OR latvia* OR lebanon* OR lebanese* OR lesotho* OR lesothan* OR lesothonian* OR basutoland* OR mosotho* OR basotho* OR liberia* OR libya* OR jamahiriya* OR lithuania* OR macedonia* OR madagasca* OR malagasy* OR malawi* OR nyasaland* OR malaysia* OR malay-federation OR malaya-federation OR malayan-federation OR maldives* OR maldivian* OR indian-ocean* OR mali* OR malta* OR maltese* OR micronesia* OR marshallese* OR kiribati* OR marshall-island* OR nauru OR nauran OR nauruans OR nauran* OR mariana OR marianas OR palau OR paluan* OR tuvalu* OR mauritania* OR mauritan* OR mauritius* OR mexico* OR mexican* OR moldova* OR moldovia* OR mongol* OR montenegr* OR morocco* OR moroccan* OR ifni OR mozambique* OR mozambican* OR myanmar* OR burma* OR burmese OR namibia* OR nepal* OR new-caledonia* OR netherlands-antill* OR nicaragua* OR niger* OR oman* OR pakistan* OR palestin* OR gaza* OR west-bank* OR panama* OR paraguay* OR peru* OR philippine* OR philipine* OR phillipine* OR phillippine* OR filipino* OR filipina*

OR poland* OR polish OR pole OR poles OR portugal* OR portuguese OR puerto-ric* OR romania* OR russia* OR ussr* OR soviet* OR rwanda* OR rwandese OR ruanda* OR ruandese OR samoa* OR navigator-island* OR pacific-island* OR polynesia* OR sao-tome* OR santomean* OR saudi-arabia* OR saudi OR saudis OR senegal* OR serbia* OR seychell* OR sierra-leone* OR slovak* OR sloven* OR melanesia* OR solomon-island* OR norfolk-island* OR somali* OR sri-lanka* OR ceylon* OR saint-kitts* OR st-kitts* OR kittian* OR nevisian* OR saint-lucia* OR st-lucia* OR saint-vincent* OR st-vincent* OR vincentian* OR grenadine* OR sudan* OR surinam* OR syria* OR tajik* OR tadjik* OR tadzhik* OR tanzania* OR tanganyika* OR thai* OR timor-leste* OR east-timor* OR timorese* OR togo* OR tonga* OR trinidad* OR tobago* OR tunisia* OR turkiy* OR turkey* OR turk OR turks OR turkish OR turkmen* OR uganda* OR ukrain* OR uruguay* OR uzbek* OR vanuatu* OR new-hebrides OR venezuela* OR vietnam* OR viet-nam* OR yemen* OR yugoslav* OR zambia* OR zimbabwe* OR rhodesia* OR arab-countr* OR arabic-countr* OR middle-east* OR global-south OR sahara* OR subsahara* OR magreb* OR maghrib* OR west-indies* OR caribbean* OR central-america* OR latin-america* OR south-america* OR central-asia* OR north-asia* OR northern-asia* OR southeastern-asia* OR south-eastern-asia* OR southeast-asia* OR south-east-asia* OR west-asia* OR western-asia* OR east-europe* OR eastern-europe* OR developing-countr* OR developing-nation* OR developing-population* OR developing-world OR less-developed-countr* OR less-developed-nation* OR less-developed-world OR lesser-developed-countr* OR lesser-developed-nation* OR lesser-developed-world OR under-developed-countr* OR under-developed-nation* OR under-developed-world OR underdeveloped-countr* OR underdeveloped-nation* OR underdeveloped-world OR middle-income-countr* OR middle-income-nation* OR middle-income-population* OR low-income-countr* OR low-income-nation* OR low-income-population* OR lower-income-countr* OR lower-income-nation* OR lower-income-population* OR underserved-countr* OR underserved-nation* OR underserved-population* OR under-served-population* OR under-served-nation* OR under-served-population* OR deprived-countr* OR deprived-population* OR high-burden-countr* OR high-burden-nation* OR countdown-countr* OR countdown-nation* OR poor-countr* OR poor-nation* OR poor-population* OR poor-world OR poorer-countr* OR poorer-nation* OR poorer-population* OR poorer-world OR developing-econom* OR less developed-econom* OR underdeveloped-econom* OR under-developed-econom* OR middle-income-econom* OR low-income-econom* OR lower-income-econom* OR low-gdp OR low-gnp OR low-gross-domestic OR low-gross-national OR lower-gdp OR lower-gnp OR lower-gross-domestic OR lower-gross-national OR lmic OR lmics OR third-world OR lami-countr* OR transitional-countr* OR emerging-econom* OR emerging-nation*):ti,ab,kw,so281965

- #5 #1 OR #2 OR #3 9842
- #6 #4 AND #5 1052
- #7 MeSH descriptor: [Adolescent] explode all trees 125417
- #8 (adolescen*):ti,ab,kw OR (child*):ti,ab,kw 293209
- #9 (teen*):ti,ab,kw 3359
- #10 (youth*):ti,ab,kw 9747
- #11 (young*):ti,ab,kw 147101

- #12 (juvenile):ti,ab,kw 4702
- #13 (schoolchild*):ti,ab,kw 1689
- #14 (schoolboy*):ti,ab,kw 29
- #15 (schoolgirl*):ti,ab,kw 75
- #16 (student*):ti,ab,kw 45927
- #17 (boy):ti,ab,kw OR (boys):ti,ab,kw 7803
- #18 (girl*):ti,ab,kw 8825
- #19 #7 OR #8 OR #9 OR #10 OR #11 OR #12 OR #13 OR #14 OR #15 OR #16 OR #17 OR #18 406518
- #20 #6 AND #19 321
- #21 MeSH descriptor: [Feeding Behavior] 2 tree(s) exploded 11741
- #22 (behavio*):ti,ab,kw 148292
- #23 (skip*):ti,ab,kw 921
- #24 (omit*):ti,ab,kw 2169
- #25 (habit*):ti,ab,kw 20829
- #26 (pattern*):ti,ab,kw 51522
- #27 #21 OR #22 OR #23 OR #24 OR #25 OR #26 214138
- #28 #27 AND #5 2107
- #29 #28 AND #4 AND #19 **121**
    - **with English filter: 118**

Web of Science

Web of Science Search Strategy (v0.1)

Database: Web of Science Core Collection

Entitlements:

- WOS.IC: 1993 to 2023
- WOS.CCR: 1985 to 2023
- WOS.SCI: 1900 to 2023
- WOS.AHCI: 1975 to 2023
- WOS.BHCI: 2005 to 2023
- WOS.BSCI: 2005 to 2023
- WOS.ESCI: 2005 to 2023
- WOS.ISTP: 1990 to 2023
- WOS.SSCI: 1900 to 2023
- WOS.ISSHP: 1990 to 2023

Searches:

1: TS=(breakfast*) Date Run: Wed Jun 28 2023 00:03:47 GMT-0400 (Eastern Daylight Time) Results: 14876

2: TS=(morning meal*) Date Run: Wed Jun 28 2023 00:03:54 GMT-0400 (Eastern Daylight Time) Results: 2153

3: #2 OR #1 Date Run: Wed Jun 28 2023 00:03:59 GMT-0400 (Eastern Daylight Time) Results: 16531

4: TS=(adolescen* OR child*) Date Run: Wed Jun 28 2023 00:04:05 GMT-0400 (Eastern Daylight Time) Results: 2677326

5: TS=(teen*) Date Run: Wed Jun 28 2023 00:04:09 GMT-0400 (Eastern Daylight Time) Results: 56699

6: TS=(youth*) Date Run: Wed Jun 28 2023 00:04:15 GMT-0400 (Eastern Daylight Time) Results: 234439

7: TS=(young*) Date Run: Wed Jun 28 2023 00:04:22 GMT-0400 (Eastern Daylight Time) Results: 1266565

8: TS=(juvenile) Date Run: Wed Jun 28 2023 00:04:28 GMT-0400 (Eastern Daylight Time) Results: 204412

9: TS=(schoolchild*) Date Run: Wed Jun 28 2023 00:05:45 GMT-0400 (Eastern Daylight Time) Results: 25029

10: TS=(schoolboy*) Date Run: Wed Jun 28 2023 00:05:53 GMT-0400 (Eastern Daylight Time) Results: 999

11: TS=(schoolgirl*) Date Run: Wed Jun 28 2023 00:06:03 GMT-0400 (Eastern Daylight Time) Results: 1469

12: TS=(boy OR boys) Date Run: Wed Jun 28 2023 00:06:35 GMT-0400 (Eastern Daylight Time) Results: 189391

13: TS=(girl*) Date Run: Wed Jun 28 2023 00:06:41 GMT-0400 (Eastern Daylight Time) Results: 214556

14: #4 OR #5 OR #6 OR #7 OR #8 OR #9 OR #10 OR #11 OR #12 OR #13 Date Run: Wed Jun 28 2023 00:07:02 GMT-0400 (Eastern Daylight Time) Results: 3964397

15: TS=(Deprived Countries OR Deprived Population OR Deprived Populations OR Developing Countries OR Developing Country OR Developing Economies OR Developing Economy OR Developing Nation OR Developing Nations OR Developing Population OR Developing Populations OR Developing World OR LAMI Countries OR LAMI Country OR Less Developed Countries OR Less Developed Country OR Less Developed Economies OR Less Developed Nation OR Less Developed Nations OR Less Developed World OR Lesser Developed Countries OR Lesser Developed Nations OR LMIC OR LMICS OR Low GDP OR Low GNP OR Low Gross Domestic OR Low Gross National OR Low Income OR Lower GDP OR lower gross domestic OR Lower Income OR Middle Income OR Poor Countries OR Poor Country OR Poor Economies OR Poor Economy OR Poor Nation OR Poor Nations OR Poor Population OR Poor Populations OR poor world OR Poorer Countries OR Poorer Economies OR Poorer Economy OR Poorer Nations OR Poorer Population OR Poorer Populations OR Third World OR Transitional Countries OR Transitional Country OR Transitional Economies OR Transitional Economy OR Under Developed Countries OR Under Developed Country OR under developed nations OR Under Developed World OR Under Served Population OR Under Served Populations OR Underdeveloped Countries OR Underdeveloped Country OR underdeveloped economies OR underdeveloped nations OR underdeveloped population OR Underdeveloped World OR Underserved Countries OR Underserved Nations OR Underserved Population OR Underserved Populations) Date Run: Wed Jun 28 2023 00:07:12 GMT-0400 (Eastern Daylight Time) Results: 1440363

16: TS=(Afghanistan OR Albania OR Algeria OR American Samoa OR Angola OR Armenia OR Azerbaijan OR Bangladesh OR Belarus OR Byelarus OR Belorussia OR Belize OR Benin OR Bhutan OR Bolivia OR Bosnia OR Botswana OR Brazil OR Bulgaria OR Burma OR Burkina Faso OR Burundi OR Cabo Verde OR Cape Verde OR Cambodia OR Cameroon OR Central African Republic OR Chad OR China OR Colombia OR Comoros OR Comores OR Comoro OR Congo OR Costa Rica OR Côte d'Ivoire OR Cuba OR Democratic People’s Republic of Korea OR Djibouti OR Dominica OR Dominican Republic OR Ecuador OR Egypt OR El Salvador OR Equatorial Guinea OR Eritrea OR Ethiopia OR Fiji OR Gabon OR Gambia OR Gaza OR Georgia OR Georgia Republic OR Ghana OR Grenada OR Grenadines OR Guatemala OR Guinea OR Guinea Bissau OR Guyana OR Haiti OR Herzegovina OR Hercegovina OR Honduras OR India OR Indonesia OR Iran OR Iraq OR Ivory Coast OR Jamaica OR Jordan OR Kazakhstan OR Kenya OR Kiribati OR Korea OR Kosovo OR Kyrgyz OR Kirghizia OR Kirghiz OR Kyrgyzstan OR Lao PDR OR Laos OR Lebanon OR Lesotho OR Liberia OR

Libya OR Macedonia OR Madagascar OR Malawi OR Malay OR Malaya OR Malaysia OR Maldives OR Mali OR Marshall Islands OR Mauritania OR Mauritius OR Mexico OR Micronesia OR Moldova OR Mongolia OR Montenegro OR Morocco OR Mozambique OR Myanmar OR Namibia OR Nepal OR Nicaragua OR Niger OR Nigeria OR Pakistan OR Palau OR Papua New Guinea OR Paraguay OR Peru OR Philippines OR Principe OR Romania OR Rwanda OR Ruanda OR Samoa OR Sao Tome OR Senegal OR Serbia OR Sierra Leone OR Solomon Islands OR Somalia OR South Africa OR South Sudan OR Sri Lanka OR St Lucia OR St Vincent OR Sudan OR Surinam OR Suriname OR Swaziland OR Syria OR Syrian Arab Republic OR Tajikistan OR Tadzhikistan OR Tajikistan OR Tadzhik OR Tanzania OR Thailand OR Timor OR Togo OR Tonga OR Tunisia OR Turkey OR Turkmen OR Turkmenistan OR Tuvalu OR Uganda OR Ukraine OR Uzbek OR Uzbekistan OR Vanuatu OR Venezuela OR Vietnam OR West Bank OR Yemen OR Zambia OR Zimbabwe) Date Run: Wed Jun 28 2023 00:07:34 GMT-0400 (Eastern Daylight Time) Results: 4145209

17: #15 OR #16 Date Run: Wed Jun 28 2023 00:07:45 GMT-0400 (Eastern Daylight Time) Results: 5230296

18: #17 AND #14 AND #3 Date Run: Wed Jun 28 2023 00:07:51 GMT-0400 (Eastern Daylight Time) Results: 1259

19: ((((TS=(behavio*)) OR TS=(skip*)) OR TS=(omit*)) OR TS=(habit*)) OR TS=(pattern*) Date Run: Wed Jun 28 2023 00:07:57 GMT-0400 (Eastern Daylight Time) Results: 8169418

20: #19 AND #3 Date Run: Wed Jun 28 2023 00:08:12 GMT-0400 (Eastern Daylight Time) Results: 5857

21: #20 AND #14 AND #17 Date Run: Wed Jun 28 2023 00:08:20 GMT-0400 (Eastern Daylight Time) Results: 899

22: #20 AND #14 AND #17 and English (Languages) Date Run: Wed Jun 28 2023 00:08:54 GMT-0400 (Eastern Daylight Time) **Results: 840**
