# Supplementary material for: Breakfast Habits, Anthropometry, and Nutrition‐Related Outcomes in Adolescents From Low‐ and Middle‐Income Countries: A Systematic Review and Meta‐Analysis
Source: Campbell Syst Rev. 2025 Apr 22;21(2):e70039. doi: 10.1002/cl2.70039 (PMC12012571; doi:10.1002/cl2.70039)

**Figure S1.** Funnel plot: Infrequent versus regular breakfast consumption on overweight/obesity outcome in adolescents from LMICs


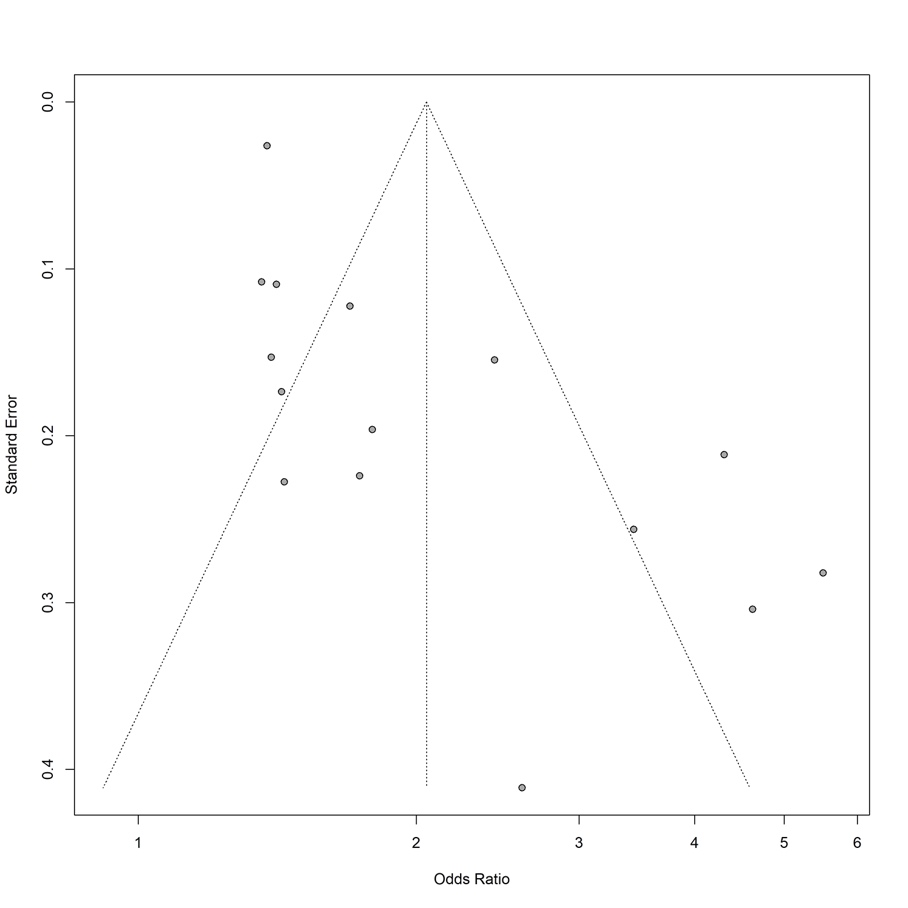


**Figure S2.** Funnel plot: Non-daily versus daily breakfast consumption on overweight/obesity outcome in adolescents from LMICs


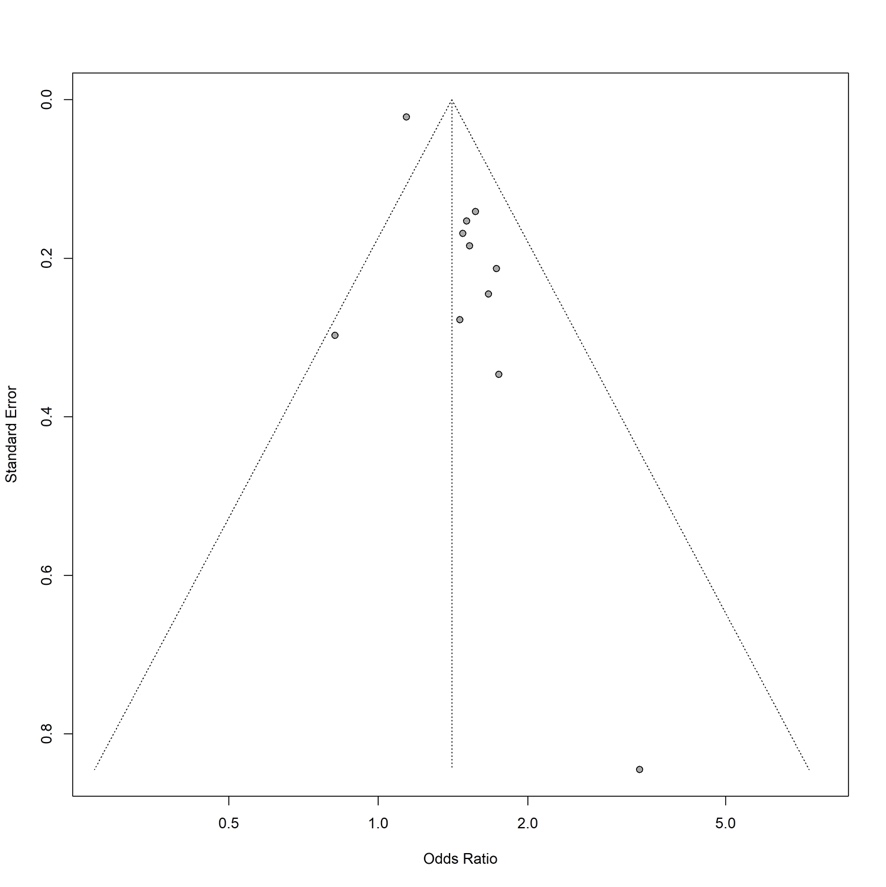


**Figures S3.** Forest plots of studies comparing odds ratio of breakfast habits on overweight (A,B,C) and obesity (D and E)

**
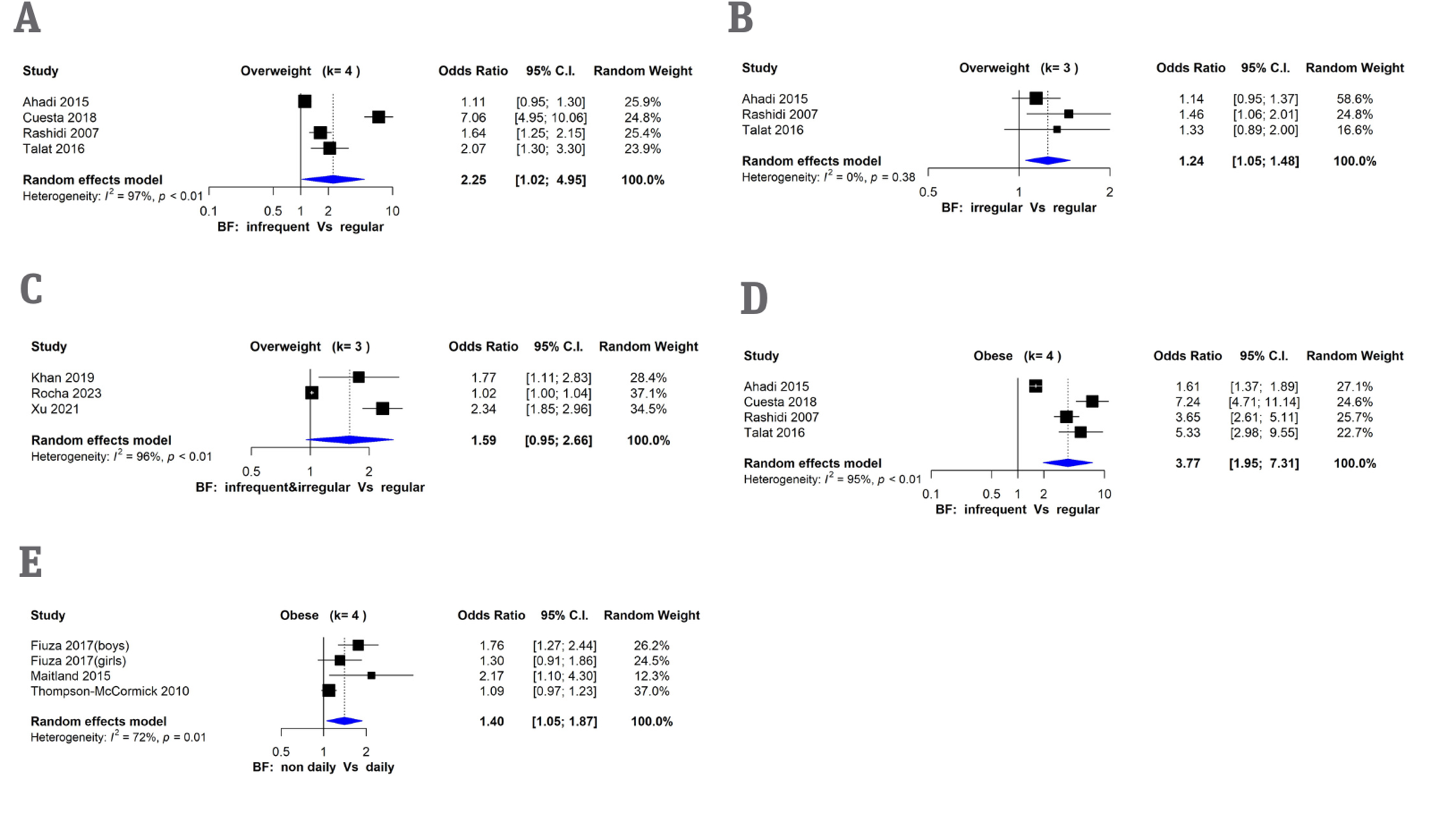
**

**Figures S4.** Forest plots of studies comparing (A and B) standardized mean difference of breakfast habits on waist circumference and (C) odds ratio of breakfast habits on elevated waist circumference.

(A)


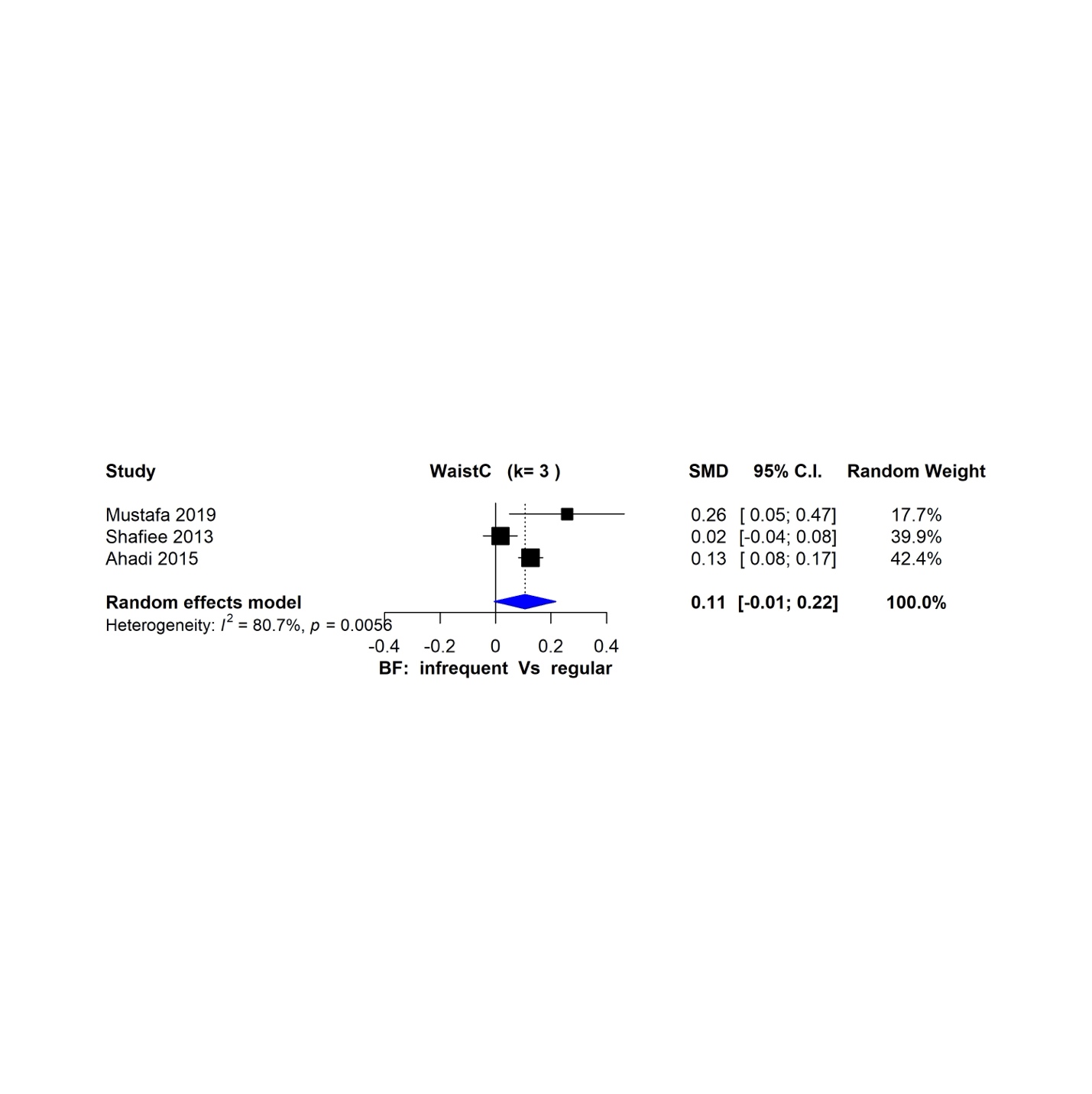


(B)


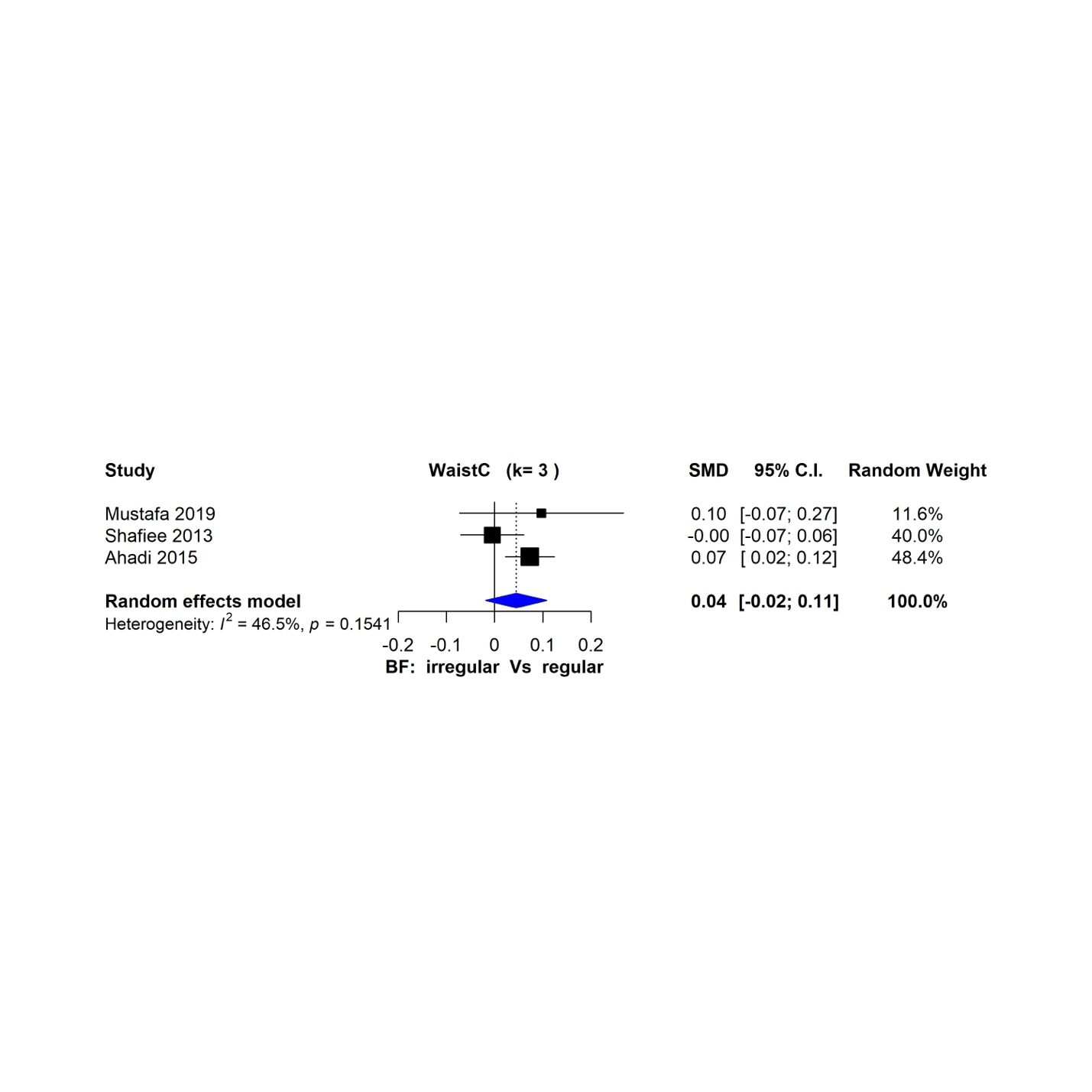


(C)


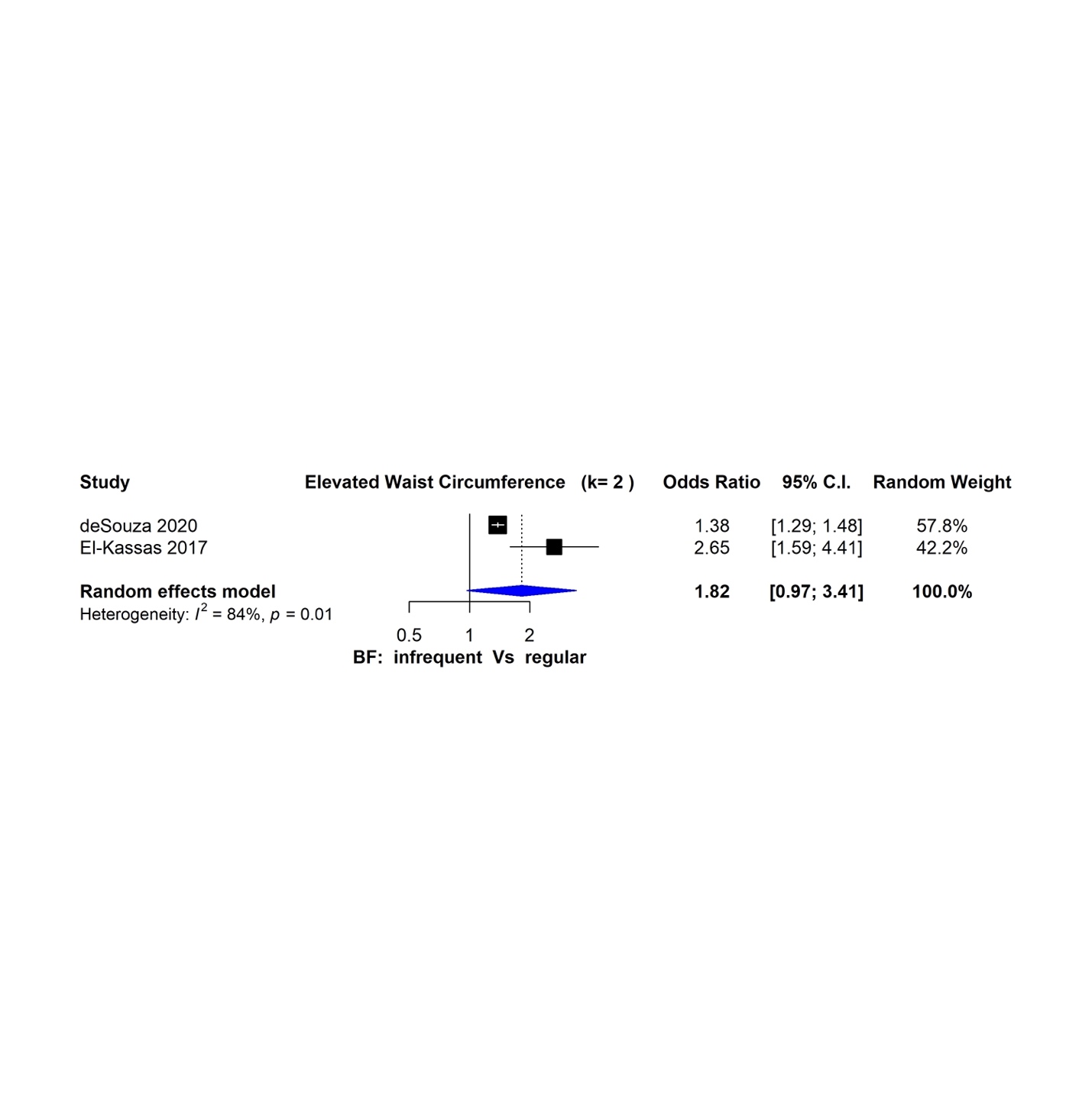


**Figures 54.** Forest plot of studies comparing (A) the odds ratio of breakfast habits on elevated waist-to-height ratio and (B, C, D) standardized mean difference of breakfast habits on elevated waist-to-height ratio

(A)


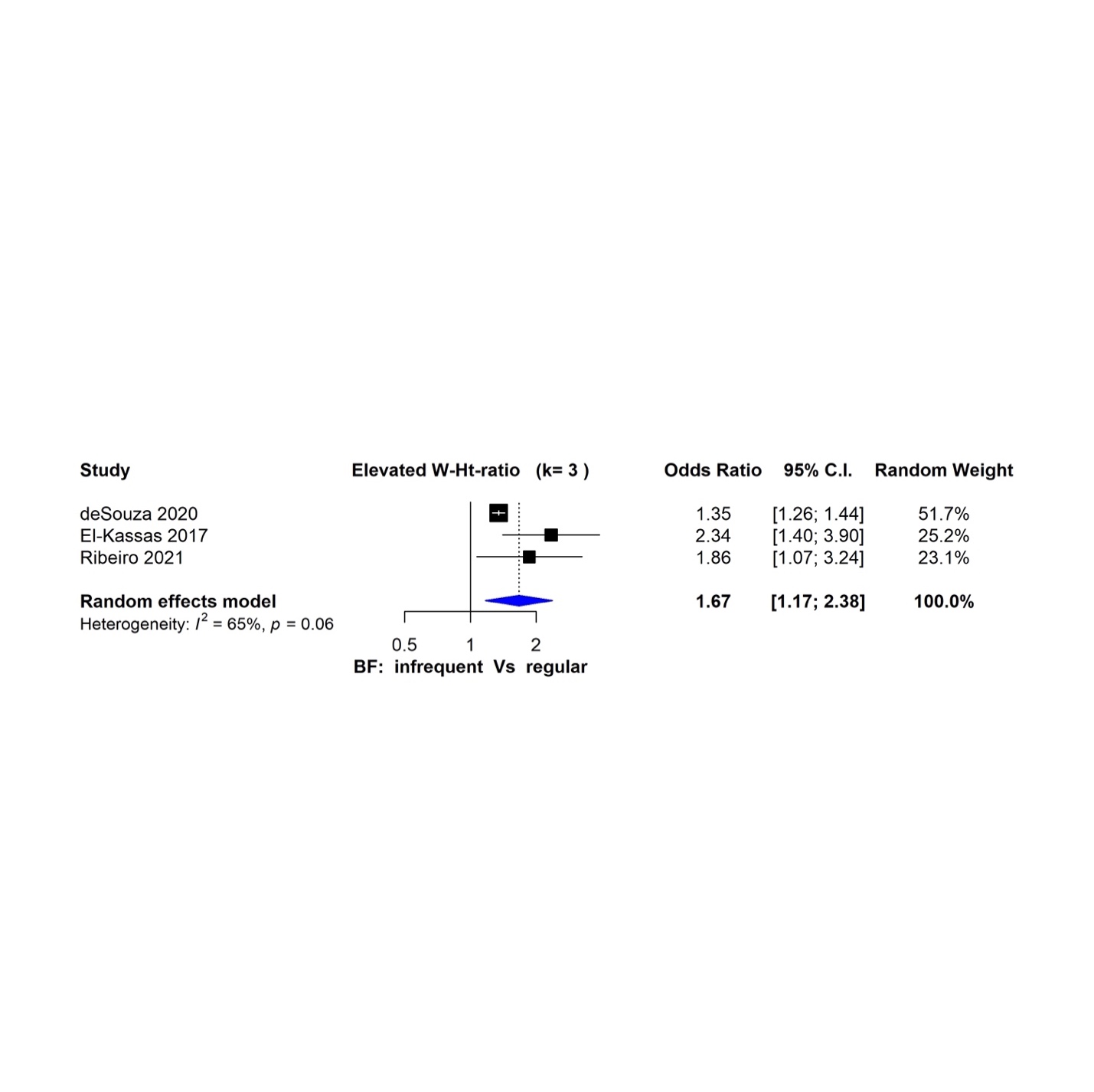


(B)


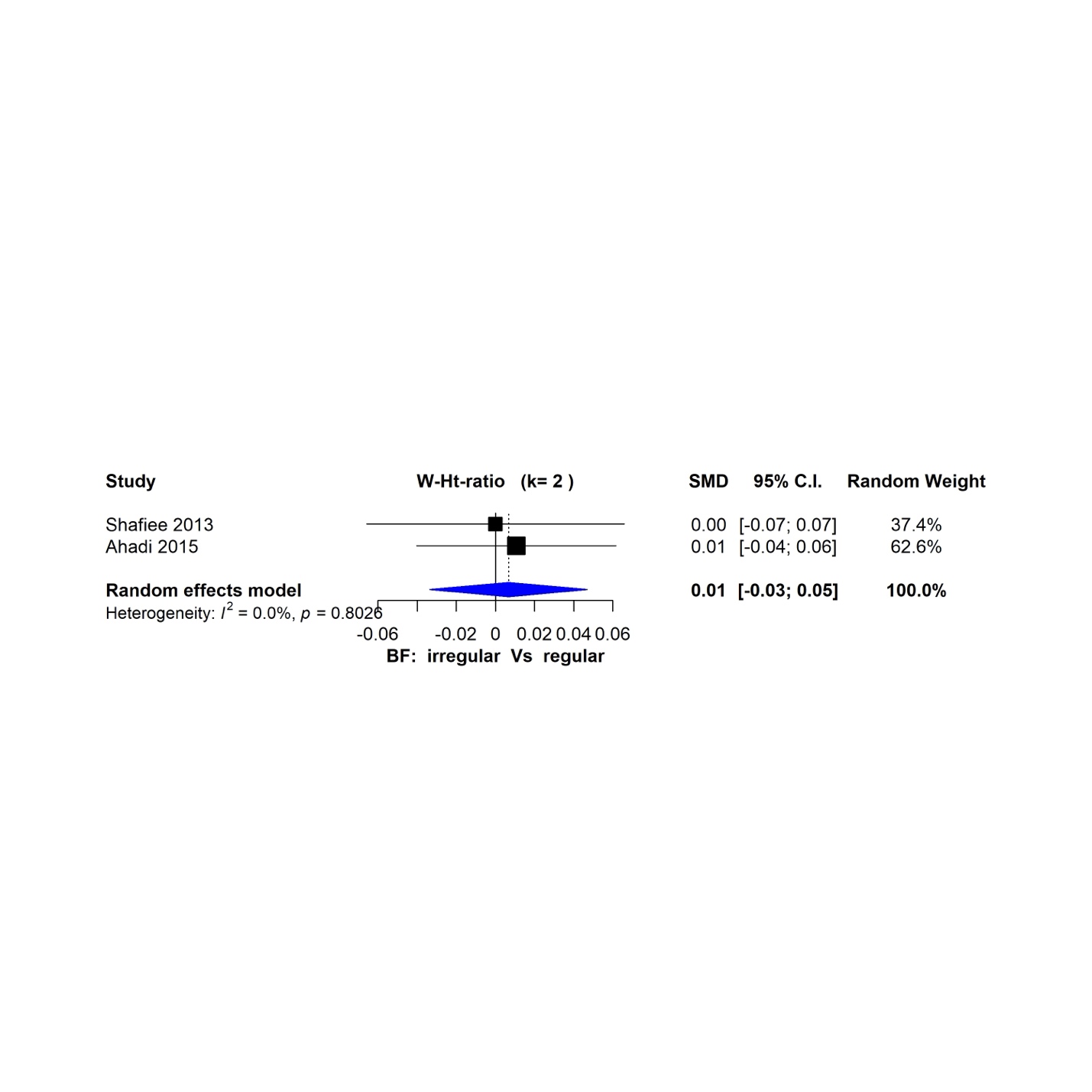


(C)


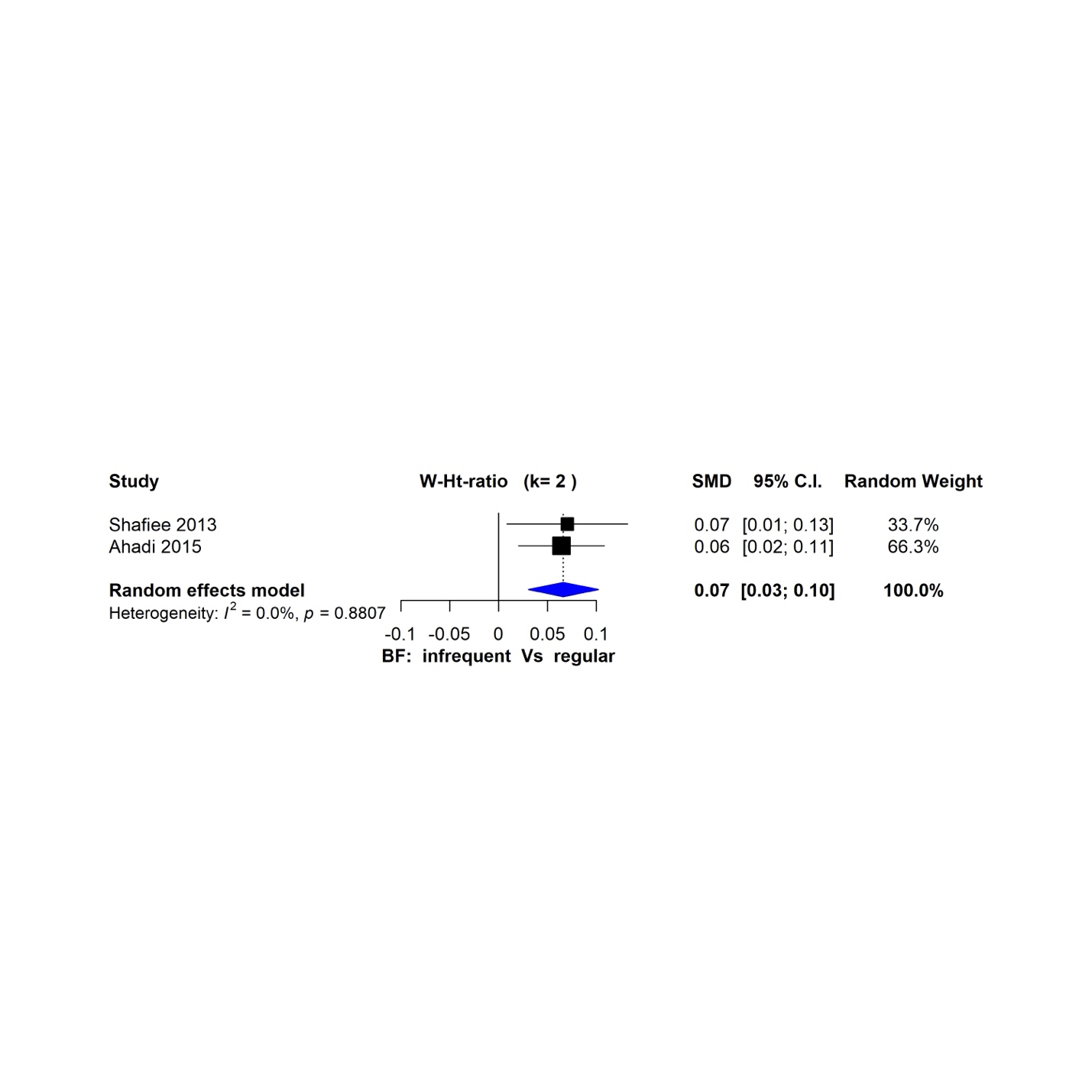


(D)


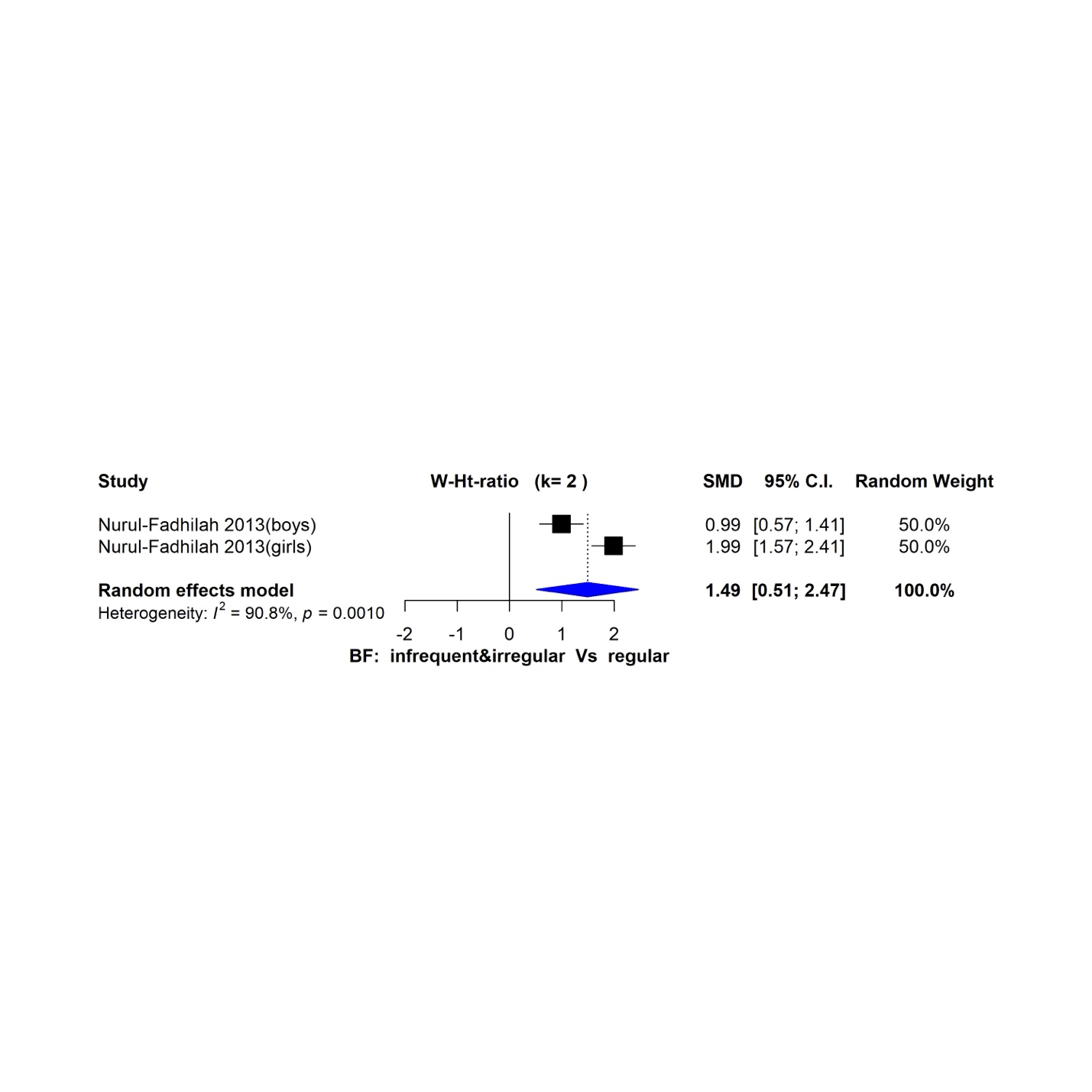


**Figures S6.** Forest plots comparing the odds ratio of breakfast habits (A - infrequent v regular; B – non-daily v daily) on overweight and obesity among studies of fair and good quality

(A)


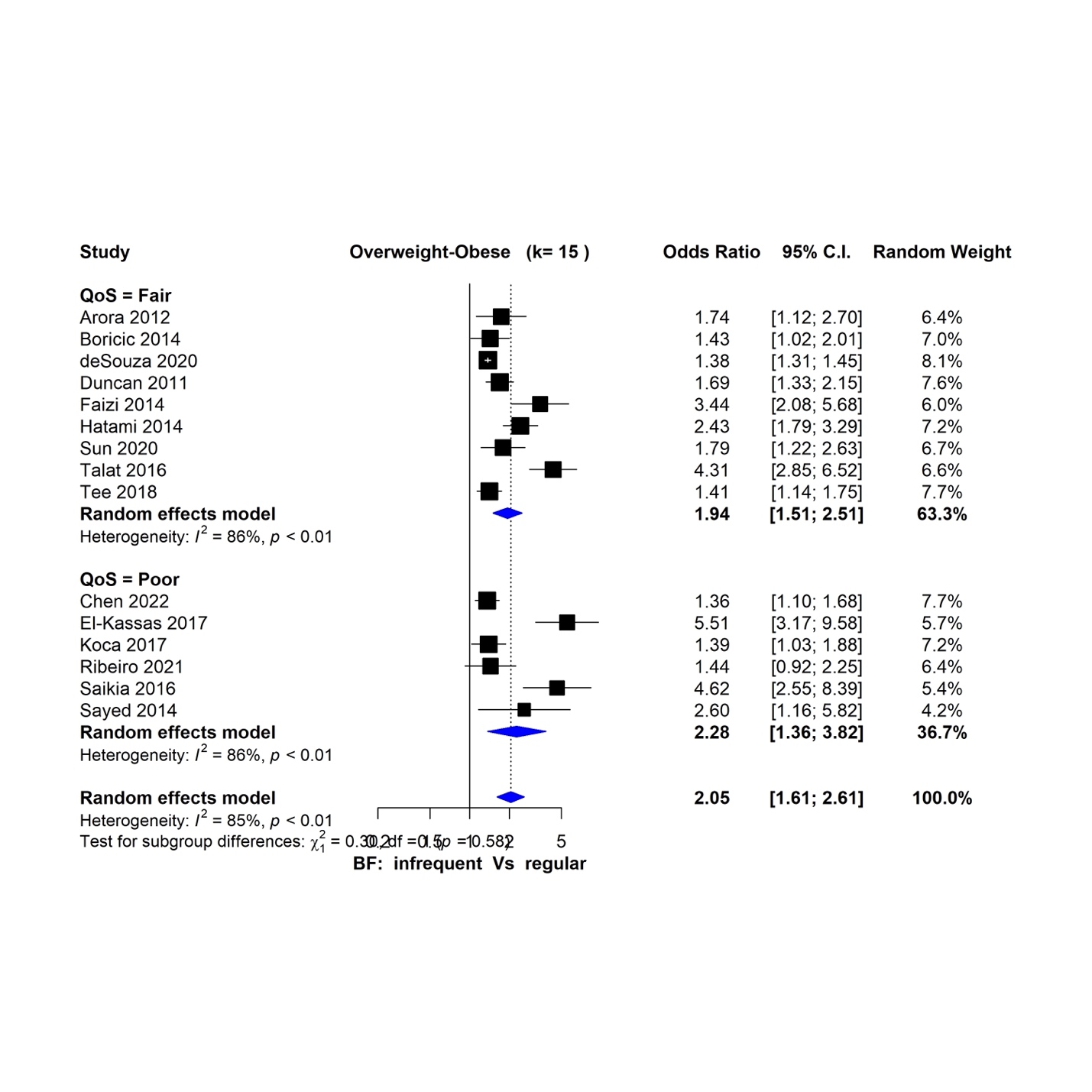


(B)


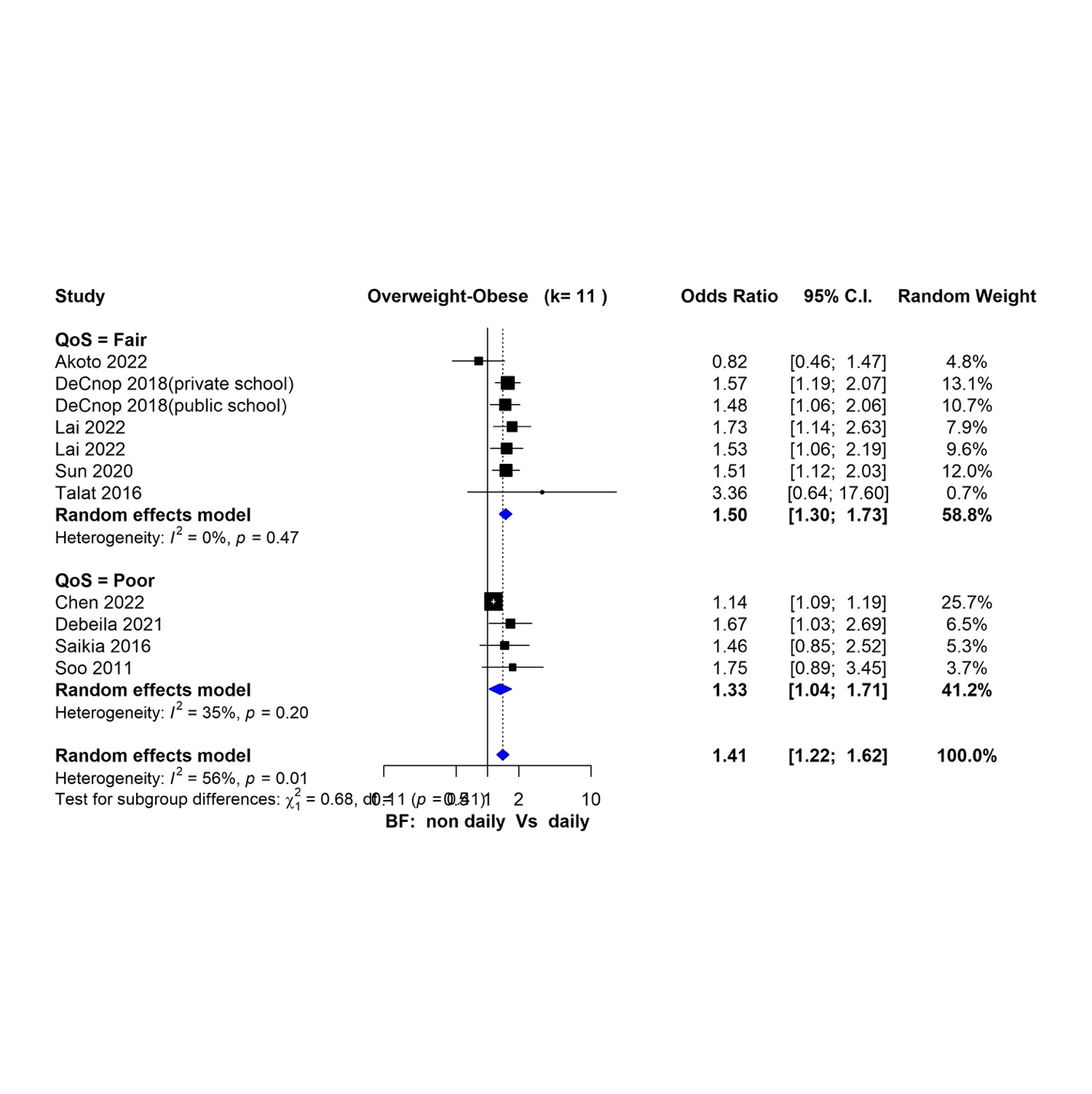


**Figures S7.** Forest plots comparing the odds ratio of breakfast habits (A and B) in adolescent boys and (C and D) girls on overweight and obesity

(A)


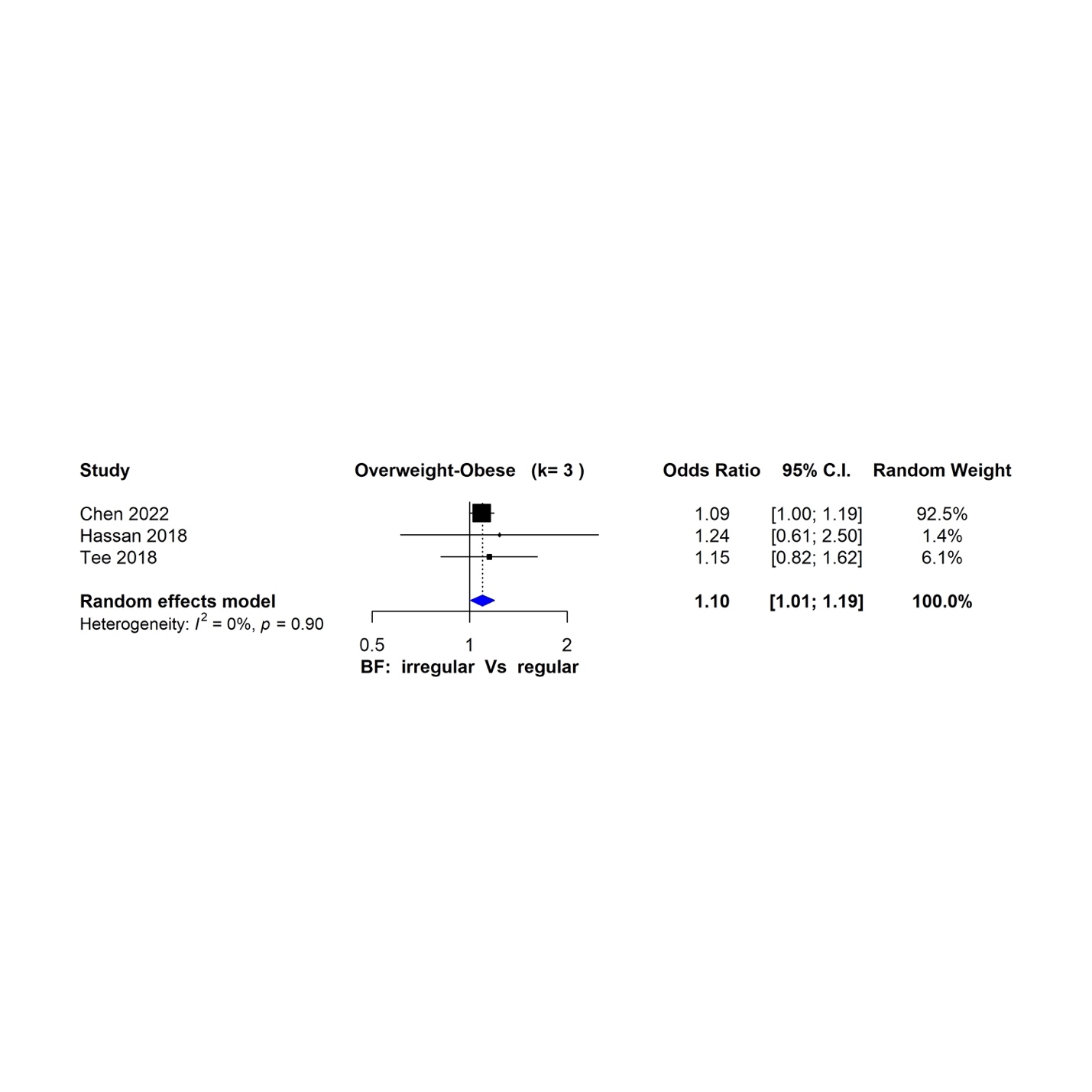


(B)


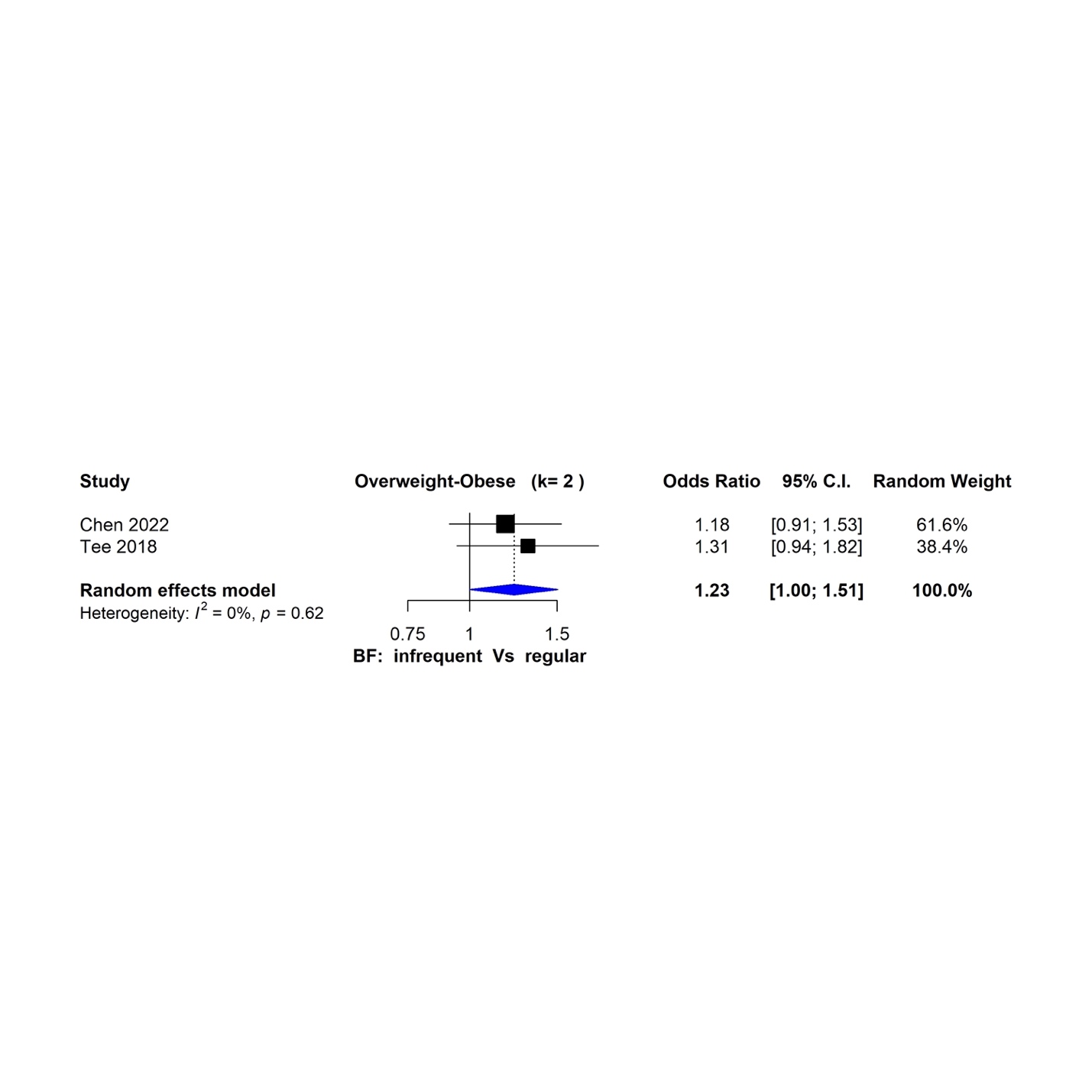


(C)


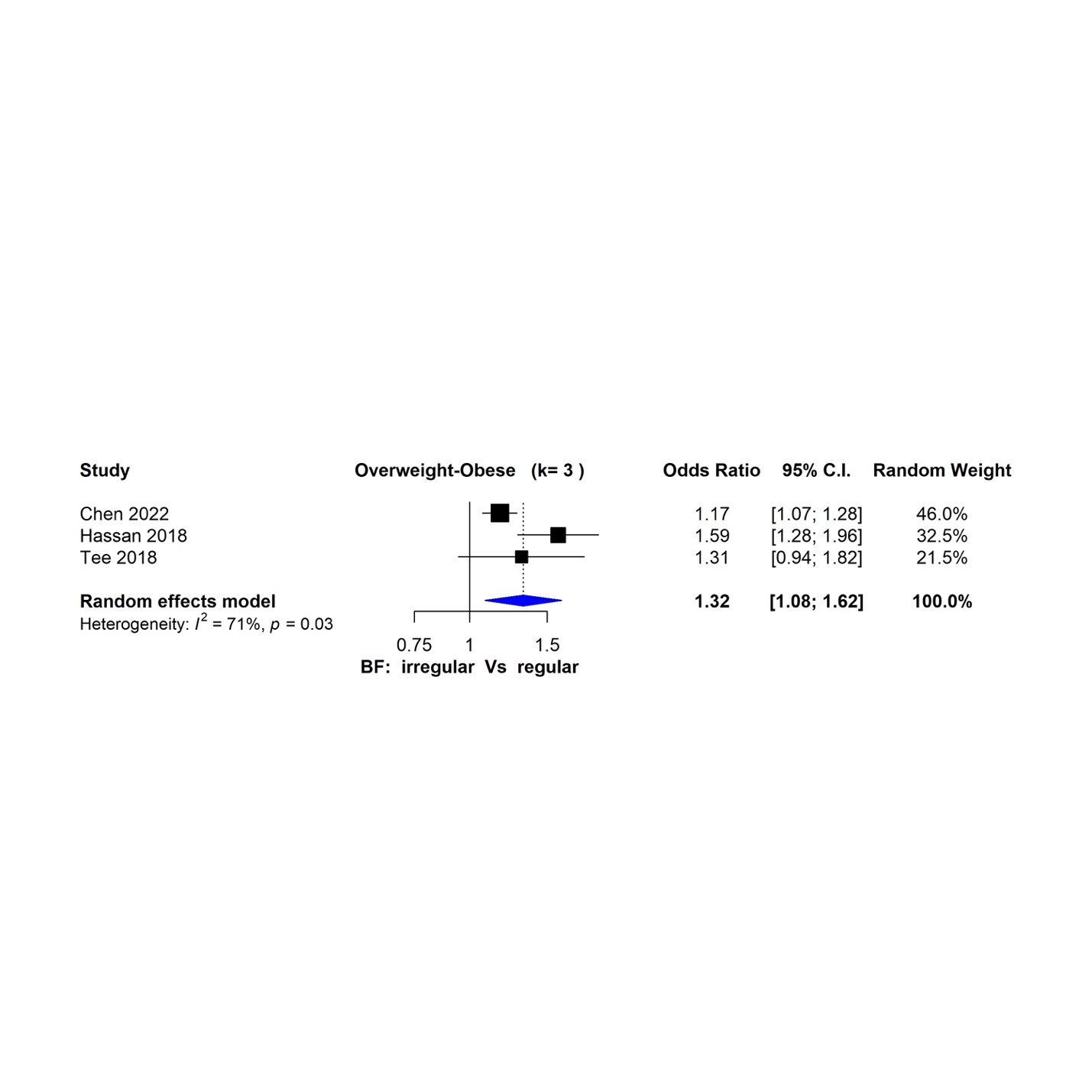


(D)


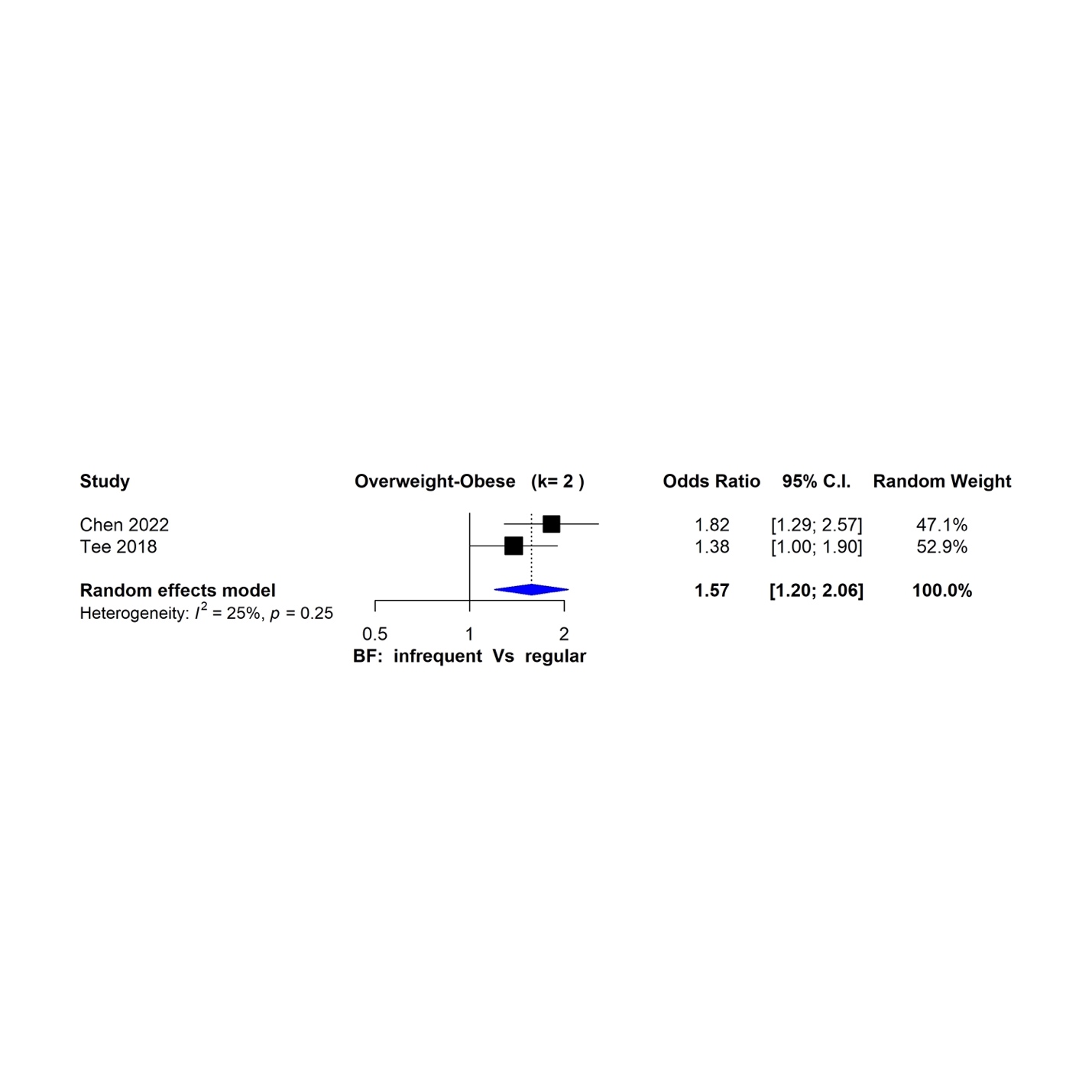

Supplement: Supplementary file 2 — Supporting information. [file CL2-21-e70039-s001.docx]
